# Two-component system ArcBA modulates cell motility and biofilm formation in *Dickeya oryzae*

**DOI:** 10.3389/fpls.2022.1033192

**Published:** 2022-10-21

**Authors:** Mingfa Lv, Sixuan Ye, Ming Hu, Yang Xue, Zhibin Liang, Xiaofan Zhou, Lianhui Zhang, Jianuan Zhou

**Affiliations:** ^1^ Guangdong Province Key Laboratory of Microbial Signals and Disease Control, Integrative Microbiology Research Centre, South China Agricultural University, Guangzhou, China; ^2^ College of Plant Protection, Fujian Agriculture and Forestry University, Fuzhou, Fujian, China; ^3^ Guangdong Laboratory for Lingnan Modern Agriculture, South China Agricultural University, Guangzhou, Guangdong Province, China

**Keywords:** *Dickeya oryzae*, two-component system, biofilm, motility, virulence

## Abstract

Phytopathogen *Dickeya oryzae* is a causal agent of rice foot rot disease and the pathogen has an array of virulence factors, such as phytotoxin zeamines, plant cell wall degrading enzymes, cell motility, and biofilms, collectively contributing to the bacterial pathogenesis. In this study, through deletion analysis of predicted regulatory genes in *D. oryzae* EC1, we identified a two-component system associated with the regulation of bacterial virulence. The two-component system contains a histidine kinase ArcB and a response regulator ArcA, and deletion of their coding genes resulted in changed phenotypes in cell motility, biofilm formation, and bacterial virulence. Electrophoretic mobility shift assay revealed that ArcA bound to the promoters of the *bcs* operon and *bssS*, which respectively encode enzymes for the synthesis of celluloses and a biofilm formation regulatory protein. ArcA could also bind to the promoters of three virulence associated transcriptional regulatory genes, i.e., *fis*, *slyA* and *ohrR*. Surprisingly, although these three regulators were shown to modulate the production of cell wall degrading enzymes and zeamines, deletion of *arcB* and *arcA* did not seem to affect these phenotypes. Taken together, the findings from this study unveiled a new two-component system associated with the bacterial pathogenesis, which contributes to the virulence of *D. oryzae* mainly through its action on bacterial motility and biofilm formation.

## Introduction

The genus of *Dickeya* is in the *Pectobacteriaceae*, which is a family of gram-negative bacteria widely distributed in water, soil, and plant tissues (Reverchon et al., 2016). *Dickeya* causes soft rot, wilts, and dwarfing diseases in a wide range of plants, including many important economic and food crops (Nasser et al., 2005; Zhou et al., 2011; Hu et al., 2018). *D. oryzae* (previously named *D. zeae*), was known previously as *Erwinia chrysanthemum* pv. *zeae* and was reclassified as a member of a new genus, *Dickeya*, in 2005 (Samson et al., 2005). It is the causal agent of maize stalk rot and rice foot rot diseases in many countries (Sinha and Prasad, 1977; Nassar et al., 1994; Samson et al., 2005; Hussain et al., 2008). Among the *Dickeya* species, *D. oryzae* is one of the members that can infect both monocotyledons and dicotyledons (Nassar et al., 1996; Samson et al., 2005; Hussain et al., 2008; Brady et al., 2012; Parkinson et al., 2014; Tian et al., 2016). Knowledge regarding the pathogenic mechanisms of the *Dickeya* genus was largely obtained through the characterization of *D. dadantii*. *D. dadantii* encodes numerous types of virulence factors, including a larger number of plant cell wall degrading enzymes (PCWDEs) (Hugouvieux-Cotte-Pattat et al., 2014), extracellular polysaccharides (EPS) (Condemine et al., 1999), the blue pigment indigotin (Wu et al., 2010), biofilms and cell motility (Jahn et al., 2008; Antunez-Lamas et al., 2009), siderophore and iron assimilation system (Enard et al., 1988; Franza et al., 2005), type I to VI secretion systems (Yang et al., 2002; Yang et al., 2008), and oxidative stress resistance (Miguel et al., 2000). Genomic analysis and biochemical characterization showed that most of these virulence traits are also conserved in *D. oryzae* (Zhou et al., 2015). In addition, a family of phytotoxins/antibiotics, named zeamine and zeamine II, were characterized as the key virulence factors of *D. oryzae* strain EC1 (Zhou et al., 2011; Cheng et al., 2013). Comparative genomic analysis showed that the zeamine biosynthetic gene cluster is conserved in the *D. oryzae* strains isolated from rice and in some strains of *D. solani* isolated from potatoes (Zhou et al., 2015). Furthermore, several lines of evidence indicate that cell motility and biofilms are also key virulence determinants of *D. oryzae*, which are driven by the putrescine quorum sensing signal (Shi et al., 2019) and the cyclic di-GMP (c-di-GMP) levels (Chen et al., 2020). In addition, cell motility was important for biofilm development and dispersion, including colonization and the expansion into mature structured surface communities (O'Toole and Kolter, 1998; Merritt et al., 2007; Holscher et al., 2015).

In recent years, several regulatory mechanisms of physiology and virulence have been characterized in *D. oryzae*. For the transcriptional factors, Fis, SlyA and OhrR have been found to regulate the production of zeamines and PCWDEs, cell motility, biofilm formation, and pathogenicity on rice seeds (Zhou et al., 2016; Lv et al., 2018; Lv et al., 2022); ExpI is responsible for the synthesis of acyl-homoserine lactone (AHL) quorum sensing signal that modulates bacterial cell motility and aggregation, and slightly affected the virulence on potato tubers (Hussain et al., 2008). For two-component system (TCS), both VfmIH and TzpSA (GacSA) have been characterized in regulation of zeamine production, cell motility, and biofilm formation (Lv et al., 2019; Chen et al., 2022). Other regulators include the Hfq encoding a conserved RNA chaperone regulating the biofilm formation and virulence to rice seeds (Shi et al., 2022), and the c-di-GMP level that mediates the capacity of cell motility and the infectivity into rice seeds (Chen et al., 2016; Chen et al., 2020). These findings reveal a valuable framework to further explore and elaborate the molecular mechanism and signaling pathway governing zeamine production and other virulence factors in *D. oryzae*.

A typical two-component system (TCS), consisting of a histidine kinase (HK) and a response regulator (RR) substrate, is one of the most common signal transduction mechanisms in bacteria. They sense various intracellular and extracellular environmental stimuli and physiological stresses through HKs, and then provide timely and appropriate responses *via* RRs in order to survive in certain environmental conditions and colonize the hosts (Ogino et al., 1998; Beier and Gross, 2006; Calva and Oropeza, 2006). TCS provides bacteria with regulation ability that controls cell motility, biofilm development, and pathogenesis (Prüß, 2017). For instance, the AtoSC TCS modulates motility and chemotactic behavior of *Escherichia coli* through transcriptional induction of the main promoters of the chemotactic regulon and the QseB (Theodorou et al., 2012; Gou et al., 2019). The PilS2R2 TCS regulates cell motility and EPS production in *Myxococcus xanthus* (Bretl et al., 2016). In *Acinetobacter baunannii*, the BfmSR, PmrAB, AdeRS, BaeSR, and GacSA TCSs are associated with its virulence, drug resistance, motility, biofilm formation, and other characteristics (Marchand et al., 2004; Tomaras et al., 2008; Adams et al., 2009; Beceiro et al., 2011; Sun et al., 2012; Cerqueira et al., 2014; Lin et al., 2014; Liou et al., 2014). In *Salmonella enteritidis*, the CpxRA TCS plays a crucial role in swarming motility and biofilm-associated phenotypes (Shetty et al., 2019). Likewise, the TCSs in *Dickeya* genus, including CpxRA, VfmIH, EnvZ/OmpR, HrpXY, PhoPQ, GacSA, play a crucial role in PCWDEs and phytotoxin production, cell motility, biofilm formation, osmoregulated periplasmic glucans (OPGs), survival and virulence to host plants (Yang et al., 2008; Yap et al., 2008; Li et al., 2009; Haque et al., 2012; Nasser et al., 2013; Bontemps-Gallo et al., 2015; Li et al., 2015; Caby et al., 2018; Lv et al., 2019; Chen et al., 2022).

Genome sequence analysis shows that *D. oryzae* EC1 contains at least 185 transcriptional factors and 74 TCSs (Zhou et al., 2015). However, the biological functions of most of these transcriptional factors and TCSs have not yet been characterized. In this study, we identified a TCS in *D. oryzae* EC1, ArcBA, whose mutation altered patterns of biofilm formation. Further functional characterization showed that ArcA (RR) can directly modulate the expression of genes involved in cellulose synthesis and biofilm formation. This TCS also plays a crucial regulatory role in the ability to infect the host, as well as swimming and swarming motility. Furthermore, we also demonstrated that ArcA can negatively regulate the expression of *slyA*, *ohrR*, and positively regulate the expression of *fis*.

## Materials and methods

### Bacterial strains and growth conditions

The bacterial strains and plasmids used in this study were listed in **Table 1**. *D. oryzae* EC1 and its derivatives were cultivated at 28°C in Luria-Bertani (LB) medium or minimal medium (MM) broth (Cheng et al., 2013) with shaking at 220 rpm, unless conditions stated. *E. coli* strains were grown at 37°C in LB medium. Antibiotics were added at the following concentrations when required: kanamycin, 50 µg/ml; polymyxin B Sulfate, 50 µg/ml; ampicillin, 100 µg/ml; streptomycin, 50 µg/ml; tetracycline, 15µg/ml.

**Table 1:** Strains and plasmids used in this study.

Strains or plasmids	Relevant phenotypes and characteristics^a^	Source or reference
Strains		
EC1	Wild type of *Dickeya oryzae*, PB^r^	Lab collection
ΔarcA	*arcA* in-frame deletion mutant derived from EC1, PB^r^	This research
ΔarcAslyA	*arcA* and *slyA* in-frame double deletion mutant derived from EC1, PB^r^	This research
ΔarcAohrR	*arcA* and *ohrR* in-frame double deletion mutant derived from EC1, PB^r^	This research
ΔarcB	*arcB* in-frame deletion mutant derived from EC1, PB^r^	This research
Δfis	*fis* in-frame deletion mutant derived from EC1, PB^r^	This research
ΔbcsA	*bcsA* in-frame deletion mutant derived from EC1, PB^r^	This research
ΔbcsB	*bcsB* in-frame deletion mutant derived from EC1, PB^r^	This research
ΔbcsC	*bcsC* in-frame deletion mutant derived from EC1, PB^r^	This research
ΔbcsD	*bcsD* in-frame deletion mutant derived from EC1, PB^r^	This research
ΔarcA(pBBR1)	Transformed the *arcA* mutant with plasmid pBBRI-MCS4, PB^r^, Amp^r^	This research
ΔarcB(pBBR1)	Transformed the *arcA* mutant with plasmid pBBRI-MCS4, PB^r^, Amp^r^	This research
ΔarcA(arcA)	Transformed the *arcA* mutant with plasmid pBBRI-MCS4 carrying the gene *arcA*, PB^r^, Amp^r^	This research
ΔarcA(arcB)	Transformed the *arcA* mutant with plasmid pBBRI-MCS4 carrying the gene *arcB*, PB^r^, Amp^r^	This research
ΔarcA(fis)	Transformed the *arcA* mutant with plasmid pBBRI-MCS4 carrying the gene *fis*, PB^r^, Amp^r^	This research
ΔarcA(ohrR)	Transformed the *arcA* mutant with plasmid pBBRI-MCS4 carrying the gene *ohrR*, PB^r^, Amp^r^	This research
ΔarcA(bcs)	Transformed the arcA mutant with plasmid pBBRI-MCS4 carrying the gene *bcs*, PB^r^, Amp^r^	This research
ΔarcA(slyA)	Transformed the *arcA* mutant with plasmid pBBRI-MCS4 carrying the gene *slyA*, PB^r^, Amp^r^	This research
ΔarcB(arcB)	Transformed the *arcB* mutant with plasmid pBBRI-MCS4 carrying the gene *arcB*, PB^r^, Amp^r^	This research
ΔbcsA(bcsA)	Transformed the *bcsA* mutant with plasmid pBBRI-MCS4 carrying the gene *bcsA*, PB^r^, Amp^r^	This research
ΔbcsB(bcsB)	Transformed the *bcsB* mutant with plasmid pBBRI-MCS4 carrying the gene *bcsB*, PB^r^, Amp^r^	This research
ΔbcsC(bcsC)	Transformed the *bcsC* mutant with plasmid pBBRI-MCS4 carrying the gene *bcsC*, PB^r^, Amp^r^	This research
ΔbcsD(bcsD)	Transformed the *bcsD* mutant with plasmid pBBRI-MCS4 carrying the gene *bcsD*, PB^r^, Amp^r^	This research
Δbcs(bcs)	Transformed the *bcs* mutant with plasmid pBBRI-MCS4 carrying the gene *bcs*, PB^r^, Amp^r^	This research
DH5α	*E. coli* strain as host for plasmid constructs derived from pBBR1-MCS4 and pLAFR3	Lab collection
HB101(pRK2013)	*Thr leu thi recA hsdR hsdM pro*, Kan^r^	Lab collection
CC118λ	*E. coli* strain as host for plasmid constructs derived from pKNG101	Lab collection
Plasmids		
pKNG101	Knockout vector, Str^r^	Lab collection
pLAFR3	Expression vector contains a *lacZ* promoter, Tc^r^	Lab collection
pBBR1-MCS4	Expression vector contains a *lacZ* promoter, Amp^r^	Lab collection
pBBR1-MCS4-arcA	pBBR1-MCS4 carries the coding region of *arcA* at down-stream of *lacZ* promoter, Amp^r^	This research
pBBR1-MCS4-arcB	pBBR1-MCS4 carries the coding region of *arcB* at down-stream of *lacZ* promoter, Amp^r^	This research
pBBR1-MCS4-bcsA	pBBR1-MCS4 carries the coding region of *bcsA* at down-stream of *lacZ* promoter, Amp^r^	This research
pBBR1-MCS4-bcsB	pBBR1-MCS4 carries the coding region of *bcsB* at down-stream of *lacZ* promoter, Amp^r^	This research
pBBR1-MCS4-bcsC	pBBR1-MCS4 carries the coding region of *bcsC* at down-stream of *lacZ* promoter, Amp^r^	This research
pBBR1-MCS4-bcsD	pBBR1-MCS4 carries the coding region of *bcsD* at down-stream of *lacZ* promoter, Amp^r^	This research
pBBR1-MCS4-fis	pBBR1-MCS4 carries the coding region of *fis* at down-stream of *lacZ* promoter, Amp^r^	Lab collection
pBBR1-MCS4-ohrR	pBBR1-MCS4 carries the coding region of *ohrR* at down-stream of *lacZ* promoter, Amp^r^	Lab collection
pLAFR3-bcs	pLAFR3 carries the coding region of *bcs* at down-stream of *lacZ* promoter, Amp^r^	This research
pLAFR3-slyA	pLAFR3 carries the coding region of *slyA* at down-stream of *lacZ* promoter, Amp^r^	Lab collection
pKNG101-ohrR	pKNG101 carries the in-frame deleted fragment of *ohrR*, Str^r^	Lab collection
pKNG101-slyA	pKNG101 carries the in-frame deleted fragment of *slyA*, Str^r^	Lab collection
pKNG101-fis	pKNG101 carries the in-frame deleted fragment of *fis*, Str^r^	Lab collection
pKNG101-ohrR	pKNG101 carries the in-frame deleted fragment of *ohrR*, Str^r^	Lab collection
pKNG101-arcA	pKNG101 carries the in-frame deleted fragment of *arcA*, Str^r^	This research
pKNG101-arcB	pKNG101 carries the in-frame deleted fragment of *arcB*, Str^r^	This research
pKNG101-bcsA	pKNG101 carries the in-frame deleted fragment of *bcsA*, Str^r^	This research
pKNG101-bcsB	pKNG101 carries the in-frame deleted fragment of *bcsB*, Str^r^	This research
pKNG101-bcsC	pKNG101 carries the in-frame deleted fragment of *bcsC*, Str^r^	This research
pKNG101-bcsD	pKNG101 carries the in-frame deleted fragment of *bcsD*, Str^r^	This research
pET32a-arcA	pET32a carries the *arcA* coding region, Amp^r^	This research
pET32a-arcB	pET32a carries the *arcB* coding region (removal of the transmembrane segments), Amp^r^	This research
pET32a-arcA_ΔREC_	pET32a carries the *arcA* coding region (removal of the REC domain), Amp^r^	This research

a pB^r^, Amp^r^, Kan^r^, Str^r^, Tc^r^ = resistance to Polymyxin B Sulfate, Ampicillin, Kanamycin, Streptomycin, or Tetracycline, respectively.

### Generation of deletion mutants and complementation strains

Strain *D. oryzae* EC1 was used as a parental strain for generation of deletion mutants using the primers listed in **Table S1**, following the methods described previously (Lv et al., 2018; Lv et al., 2019). For complementation, the coding regions of the genes were amplified by PCR using the primers listed in **Table S1** and cloned in expression vectors pBBR1-MCS4 and pLAFR3 as indicated. The resultant constructs were transferred into *D. oryzae* deletion mutants through triparental mating.

### Measurement of bacterial growth kinetics

*D. oryzae* strains EC1, ΔarcA, ΔarcB, ΔarcA(arcA) and ΔarcB(arcB) were grown in LB medium about OD_600 =_ 1.5, and 500 µL of bacterial solution was added into the erlenmeyer flask containing 200 ml of LB and SOBG liquid media. LB and SOBG media (per litre contains tryptone 20 g, yeast extract 5 g, MgSO_4_ 1.2 g, NaCl 0.5 g, KCl 0.186 g, and glycerol 20 ml) (Yap et al., 2005) were set as the blank controls. The cell density of the bacterial cultures was measured every 4 h for a total of 48 h. The experiment was repeated three times.

### Biofilm formation assay

Non-adherent biofilms at air/liquid interface were measured as the following: bacterial cultures were grown overnight in LB medium and diluted in SOBG (Yap et al., 2005) medium to a density at OD_600_ = 0.01 and an aliquot of 3 ml bacterial dilutions was added into each glass tube (15×100 mm) and statically cultured at 28°C for 48 h. Photographs were taken by a SONY camera.

Attached biofilms were measured as described previously (Lv et al., 2018; Lv et al., 2022). Bacterial cultures were grown overnight in LB medium and diluted in SOBG medium to a density at OD_600_ = 0.01 and an aliquot of 100 μl bacterial dilutions was added into each well of 96-well microlitre plate and incubated at 28°C with shaking at 150 rpm for 18 h. The liquid cultures were removed and 150 μl of 1% crystal violet (wt/vol) were added to each well, after staining at room temperature for 15 min. The dye solutions were removed and the wells were washed three times with distilled water and drying under open air. The wells were added with 200 μl of 95% ethanol to dissolve the dye and quantitative determination of the crystal violet was performed by measuring the spectrophotometric values at 595 nm with a microplate reader (BioTek).

### Swimming and swarming motility assay

For measuring the swimming motility of *D. oryzae* strains, the plates were prepared by pouring 15 ml of semisolid swimming medium (per litre contains 10 g Bacto tryptone, 5 g NaCl, and 2 g agar) into the 90 mm petri dish, and spotted with 1µl bacterial dilutions grown overnight in LB medium (OD_600_ = 1.0) and incubated at 28°C for 18 h. The diameter of bacterial zone was measured. The swarming motility was assayed in the same condition except the medium (per litre contains peptone5 g, yeast extract 3 g, and agarose 4 g) and different incubation time at 14 h. The experiment was repeated three times with triplicates each time.

### Analysis of oxidative stress resistance

*D. oryzae* strain EC1 and deletion mutants of ΔarcA and ΔarcB were grown in LB medium to OD_600_ = 1.5 ± 0.05. Bacterial cultures (1.5 μl) were inoculated into each well of 96-well microtiter plate containing 150 μl fresh LB medium, which contained hydrogen peroxide at a final concentration of 0.1-1.0 mM with four replicates per treatment and two repeats. The plate was incubated at 28 °C with shaking at 150 rpm for 18 h. The optical density at 600 nm of bacterial culture was measured by a microplate reader (BioTek).

### Determination of plant cell wall degrading enzymatic activities

Cellulase (Cel), pectate lyase (Pel), polygalacturonase (Peh) and proteolytic (Prt) enzymatic activities were determined using carboxymethyl cellulose sodium, polygalacturonic acid and skimmed milk as substrates, respectively, following the methods described previously (Chatterjee et al., 1995; Caldas et al., 2002). The enzymatic activities were measured as follows: assay plate was prepared by pouring about 35 ml of substrate medium into the 120 × 120 mm petri dish, and wells of 5 mm in diameter were punched in the assay plate after solidification; bacteria were cultured overnight at 28°C in LB medium when the population density reached about OD_600_ = 1.4; 20 μl of the supernatants were taken and added into the wells of the assay plate (Chatterjee et al., 1995), and incubated at 28°C. The Cel assay plates incubated for 14 h were stained with 0.1% Congo red (w/v) for 10 min and then decolored with 1M NaCl for 15 min three times. The Pel and Peh plates were treated with 1 M HCl for coloration after 11 h post incubation under the same temperature. The transparent zones surrounding the wells of the Prt assay plates were recorded after incubation for 24 h. The experiment was repeated three times with triplicates.

### Measurement of antimicrobial activity and quantification of zeamines

The antimicrobial activity bioassay plates were prepared by pouring 15 mL of LB agar medium into the 120 × 120 mm plates, and then overlaid with 20 mL of 1% agarose containing 1.0 × 10^8^ cells of fresh *E. coli* DH5α. Wells of 5 mm in diameter were punched after solidification. Overnight bacterial cultures were grown in LS5 medium (Liao et al., 2014) to OD_600_ at around 1.4, and 20 μl of supernatants were taken and added into the wells of the assay plates. The plates were incubated at 37°C for 18 h. The antimicrobial activity was determined by measuring the radii of the visible clear zones surrounding the wells. The concentration of zeamines was determined by this formula: zeamines (unit) = 0.5484e^0.886^ *^x^*, the correlation coefficient is 0.9957 and *x* is the radius in millimeters of the inhibition zones surrounding the wells (Liao et al., 2014; Lv et al., 2018; Lv et al., 2022).

### Rice seed germination and bacterial invasion assays

The rice seed (cv. Texianzhan, from the Rice Research Institute, Guangdong Academy of Agricultural Sciences, Guangzhou, China) germination assay was performed as previously described (Lv et al., 2022). Briefly, twenty seeds were added to 5 mL of ultrapure water containing 10^2^ cells and incubated at room temperature for 5 h. The rice seeds were then washed three times with sterilized water and transferred onto two moistened filter papers in a petri dish. The seeds were then incubated at 27°C with a 16-h light and 8-h dark cycle, and sterilized water was added when necessary. Rice seeds were incubated with the same amount of sterilized water as a blank control. The rate of seed germination was determined one week after treatment. The experiment was repeated three times.

To visualize bacterial invasion, the encoding region of *gfp* was amplified by PCR, and cloned under the control of the *lac* promoter in the expression vector pLAFR3, which carries a tetracycline resistance gene. The resultant construct was introduced into *D. oryzae* strain EC1 and mutant ΔarcA *via* triparental mating. Overnight cultures of GFP labeled strains EC1 and ΔarcA were resuspended and diluted with ddH_2_O to 10^2^ CFU. Twenty rice seeds were added to 1 ml of bacterial dilution and incubated at room temperature for 5 h, and then transferred onto moistened filter papers in plates. Rice seeds were incubated with the same amount of sterilized water as a blank control. The seeds were incubated at 28 °C in 16-h light and 8-h dark conditions for 24 h. The husks of rice seeds were removed and then examined under a fluorescence microscope.

### RNA extraction, purification, and reverse transcription-quantitative PCR (RT-qPCR)

RNA samples were isolated from fresh bacterial cultures (OD_600_ = 1.0) using the SV total RNA isolation system kit (Promega). The integrity of RNA was visualized by agarose gel electrophoresis and the concentration of RNA was measured using a NanoDrop ND-100 spectrophotometer.

Reverse transcription PCR was performed using a HiScript III SuperMix for qPCR (Vazyme) according to the manufacturer’s instructions. Specific RT-qPCR primers listed in **Table S1** were used to amplify central coding fragments of approximately 200 bp in length from different genes. The quality of primers for amplification capability was determined by the melting curve analysis. SYBR Green qPCR Master Mixes (Vazyme) was used according to the manufacturer’s instructions. As a control, RT-qPCR was similarly performed to analyze *arcA* gene expression. The absolute value of −ΔΔ*C*_t_ = −(Δ*C*_t1_ − Δ*C*_t2_) was calculated as described in the formula 2^−ΔΔ^ *^C^* ^t^ (Livak and Schmittgen, 2001). The RT-qPCR experiment was repeated at least twice and the cDNA samples were prepared from triplicate cultures each time.

### Electrophoretic motility shift assay (EMSA)

The prokaryotic protein expression vector pET32a was linearized by *Bam*HI and *Hind*III. The coding sequences of *arcA* and *arcB* (removal the sequences of the transmembrane segments) were amplified using primers pET32a-arcA/arcB-BamHI-F and pET32a-arcA/arcB-HindIII-R (**Table S1**), respectively. The resultant 717-bp DNA fragment of *arcA* and 2,100-bp DNA fragment of *arcB* coding regions were cloned into the *Bam*HI-*Hind*III digested vector pET32a by ClonExpress MultiS (Vazyme) to generate pET32a-arcA and pET32a-arcB (**Table 1**). The ArcA-His and ArcB-His proteins were induced and purified following the method described previously (Lv et al., 2019). The *E. coli* BL21 cells containing pET32a-arcA and pET32a-arcB were induced to express ArcA and ArcB by adding IPTG to a final concentration of 0.5 mM at 18°C overnight. The proteins were purified by affinity chromatograph following the procedure of HisTALON™ Gravity Column Purification Kit User Manual (Clontech) and stored at -80°C. The expression of the REC-deleted ArcA protein was also performed using the above method.

The DNA sequences of target promoter regions were amplified using the primers listed in **Table S1**. The purified PCR products were labeled by biotin using the Biotin 3’ End DNA Labeling Kit (Thermo). The reaction mixture contained 20 fmol labeled DNA fragments and 0 μM, 1 μM, 3 μM or 6 μM ArcA protein as indicated in a final volume of 10 μl. The protein-DNA complexes and the unbound free DNA fragments were separated on 6% nondenaturing polyacrylamide (acrylamide/bisacrylamide 29:1 v/v) gel using the electrophoresis buffer TBE, and detected by chemiluminescence (Tanon). The specific interaction of ArcA protein-DNA fragments was verified by incubation of 100-fold molar excess of unlabeled DNA fragments with ArcA protein before the addition of labeled DNA fragments.

For verification of the interaction between ArcB and ArcA, the reaction mixture contained 30 ng of DNA fragments, 0 μM, 0.3 μM, 0.6 μM, 0.9 μM or 1.2 μM of ArcA/REC-deleted ArcA, and 0.5 μM of ArcB protein as indicated in a final volume of 10 μl. The free DNA and the protein-DNA complexes were separated on 5% nondenaturing polyacrylamide (acrylamide/bisacrylamide 29:1 v/v) gel using the electrophoresis buffer TAE, and detected by gel imaging system (Tanon 3500).

### Statistical analysis

Each experiment was performed with triplicates and repeated at least three times unless otherwise indicated. For easy comparison, certain data of mutants were normalized to those of the wild-type EC1, which were arbitrarily set as 100%. The paired two-tailed Student’s *t* test and significantly different values (analysis of variance, *p* < 0.05) were performed between the wild-type EC1 and its derivatives using Prism v. 8.0 software (GraphPad).

## Results

### Deletion of *arcB* and *arcA* decreased the biofilm formation in *D. oryzae* strain EC1

**Figure 1:**
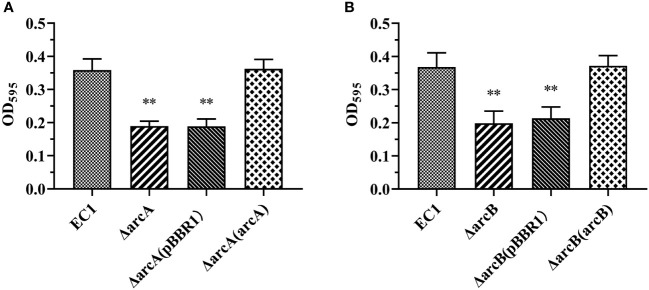
The deletion mutants of *arcA* and *arcB* shown decreased capacity for biofilm formation compared with *D*. *oryzae* wild-type strain EC1. **(A)** Quantified adhesion biofilm biomass of wild-type EC1, ΔarcA, ΔarcA(pBBR1) and complemented strain ΔarcA(arcA) using crystal violet staining. **(B)** Quantified adhesion biofilm biomass of wild-type EC1, ΔarcB, ΔarcB(pBBR1) and complemented strain ΔarcB(arcB) using crystal violet staining. The experiments were repeated three times in triplicates. **p < 0.01, Student’s *t* test.

To identify the genes that regulate virulence in addition to SlyA, Fis, VfmIH, and OhrR in *D. oryzae* EC1 (Zhou et al., 2016; Lv et al., 2018; Lv et al., 2019 and Lv et al., 2022), we generated deletion mutants of eight selected genes that were predicted to encode transcriptional regulators belonging to different regulator families (a SlmA, two MarR, two AcrR, a DctR, and a Fnr) and a TCS RR (ArcA) in EC1 genome and examined their biofilm formation (**Table S2**). The ArcA TCS RR contains 238 amino acids and shares 94% protein sequence identity with the previously characterized *E. coli* ArcA (RefSeq id: NP_418818.1) (**Figure S1A**), whose deletion reduced the biofilm formation in *E. coli* (Jiang et al., 2015). ArcA contains a REC domain at its N-terminal and a Trans_reg domain at the C-terminal (**Figure S1C**). Deletion of *arcA* resulted in significantly decreased biofilm formation compared with the wild-type strain EC1 (**Figure 1A**). Sequence alignment of the cognate histidine kinase ArcB from *E. coli* (RefSeq id: YP_026207.1) led to identification of a homolog gene (gene locus as *W909_RS01585*) in strain EC1 genome, which encodes a peptide of 779 amino acids sharing about 74% identity at amino acid level with its counterpart in *E. coli* (**Figure S1B**). ArcB contains two transmembrane (TM) domains, a PAS, a PAC, a HisKA, a HATPase_c, a REC and a HTP domains (**Figure S1C**). Consistently, deletion of *arcB* also resulted in markedly decreased biofilm formation compared with its parental strain EC1 (**Figure 1B**). This alteration in biofilm formation was irrelated to the growth difference between strains at the exponential growth phase, although mutation of *arcA* and *arcB* decreased the cell density at the stationary phase (**Figure S2**). Transformation of ΔarcA and ΔarcB mutants with pBBR1 plasmids carrying the wild-type *arcA* and *arcB* genes, respectively, restored the biofilm formation to the wild-type strain EC1 level (**Figures 1A, B**), while those carrying the empty vector pBBR1 showed similar biofilm formation to the mutants ΔarcA and ΔarcB (**Figures 1A, B**).

### Deletion of *arcB* and *arcA* did not affect the production of plant cell wall degradation enzymes and zeamines

Plant cell wall degradation enzymes (PCWDEs) and zeamines are two families of virulence factors produced by EC1. To investigate the potential involvement of ArcBA in regulation of these virulence factors, we tested the enzymatic activities and the growth inhibitory activities against *E. coli* DH5α of strain EC1 and its deletion mutants of ΔarcA and ΔarcB, and found that neither mutant was impaired in these functions (**Figures S3**, **S4**).

### ArcBA modulates the oxidative stress resistance mechanism in *D. oryzae* EC1

**Figure 2:**
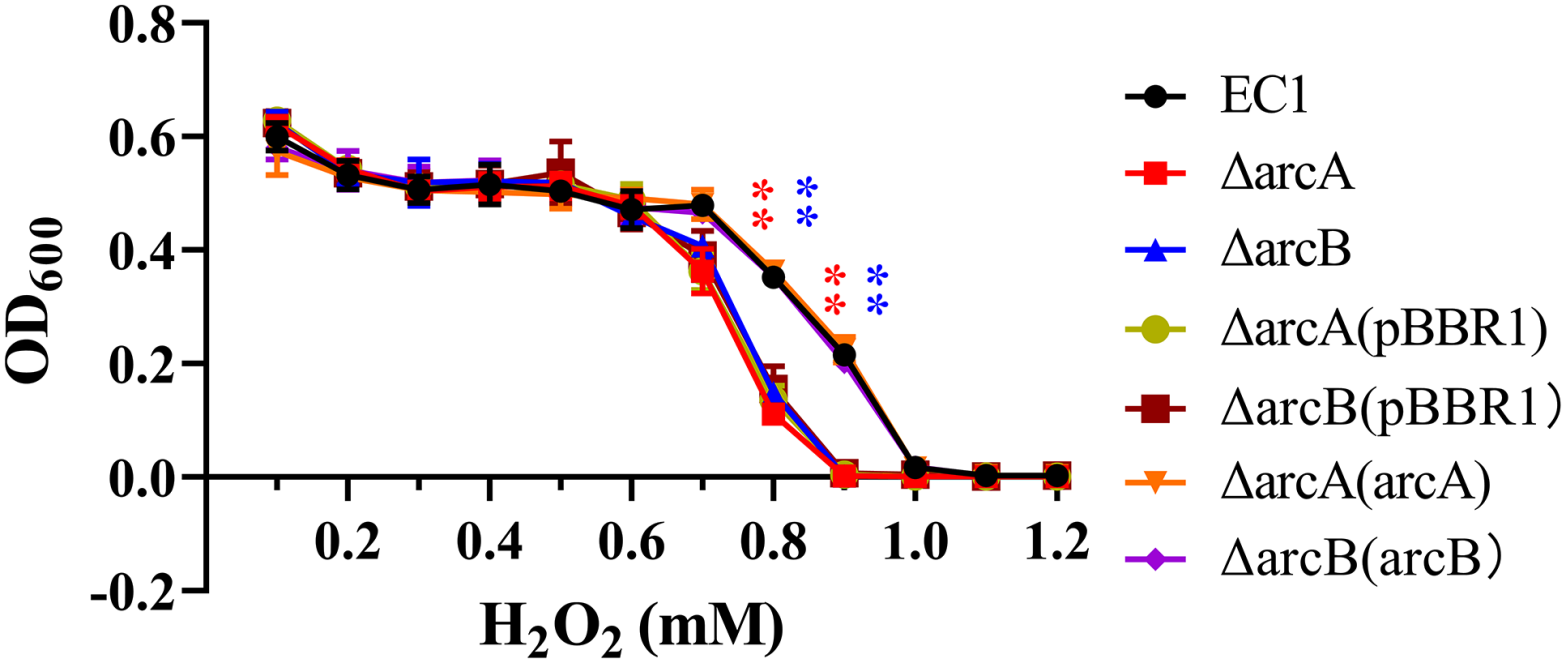
The deletion mutant of *arcA* and *arcB* enhanced the sensitivity to hydrogen peroxide. Both strain EC1, mutants ΔarcA, ΔarcB, ΔarcA(pBBR1) and ΔarcB(pBBR1), complemented strains ΔarcA(arcA) and ΔarcB(arcB) were inoculated in LB medium containing different final levels of H_2_O_2_ as indicated and measured at 600 nm using a microplate reader (BioTek). Three independent experiments were performed in triplicate. **p < 0.01, Student’s *t* test.

In the process of establishing infection, microbial pathogens have to overcome various host defense mechanisms. One of the well-established defense mechanisms that host plants deployed to withstand microbial infections is the rapid production and accumulation of reactive oxygen species (ROS) at the infection court, known as oxidative burst (Yoshioka et al., 2009; Singh et al., 2021). To determine whether ArcBA is involved in modulation of the *D. oryzae* resistance against the ROS generated by host plants, we examined the sensitivity of mutant ΔarcA and ΔarcB to hydroperoxide (H_2_O_2_), which is a common ROS species produced by host plants during host-pathogen interaction (Yoshioka et al., 2009). The assay results showed that ΔarcA and ΔarcB mutants and their complemented strains with the empty pBBR1 vector became more sensitive to hydroperoxide than the wild type EC1 and the complemented strains ΔarcA(ArcA) and ΔarcB(ArcB) when the hydroperoxide concentration was in the range of 0.7-0.9 mM (**Figure 2**). This result suggested that the ArcBA TCS plays a role in regulation of ROS resistance in *D. oryzae*.

### Deletion of *arcB* and *arcA* reduced bacterial motility

**Figure 3:**
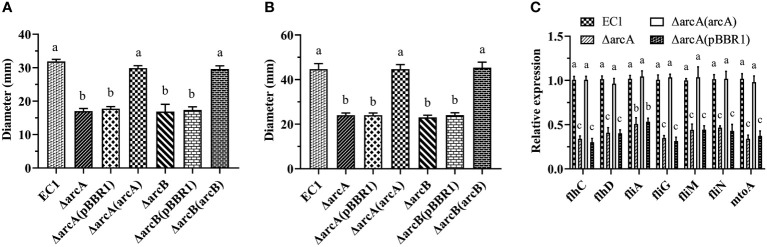
Deletion mutants ΔarcA and ΔarcB showed decreased bacterial swimming **(A)** and swarming **(B)** motility compared with wild-type strain EC1 and their complementary strains. The overnight bacterial culture (1 μl) was spotted on the center of the bacterial motility plates and incubated at 28 °C for 18 h (swarming motility) and 14 h (swimming motility) before measurements. **(C)** Gene expression of flagellar genes *flhC*, *flhD*, *fliA*, *fliG*, *fliM*, *fliN* and *motA* in strains ΔarcA and ΔarcA(pBBR1). Each experiment was repeated three times in triplicate. The statistical analysis was performed on each group of data, and significantly different values (analysis of variance, *p* < 0.05) are indicated by different letters.

Swimming and swarming are two different types of bacterial motility that play a role in their territorial aggression and systemic infection. To understand the role of ArcBA in *D. oryzae* motility development, we tested whether deletion of *arcA* and *arcB* might affect the bacterial swimming and swarming motility. The results showed that both the swimming and swarming motility of the ΔarcA and ΔarcB mutants and their empty vector complemented strains ΔarcA(pBBR1) and ΔarcB(pBBR1) was significantly reduced compared with the wild type EC1 (**Figures 3A, B**). *In trans* expression of *arcA* and *arcB* in the corresponding mutant strains restored the swimming and swarming motility to the level of wild-type strain EC1 (**Figures 3A, B**). In addition, we also tested the expression of the flagellar transcriptional regulator encoding genes *flhC* and *flhD*, an RNA polymerase sigma factor encoding gene *fliA*, flagellar motor switch protein encoding genes *fliG*, *fliM* and *fliN*, and a flagellar motor stator protein encoding gene *motA* by RT-qPCR. Results showed that their expression were decreased markedly in ΔarcA and ΔarcA(pBBR1) mutants compared with the wild-type strain EC1 and the complemented strain ΔarcA(arcA) (**Figure 3C**), validating the key role of ArcBA in positive regulation of bacterial motility.

### Deletion of *arcA* significantly decreased the expression of cellulose synthesis genes

**Figure 4:**
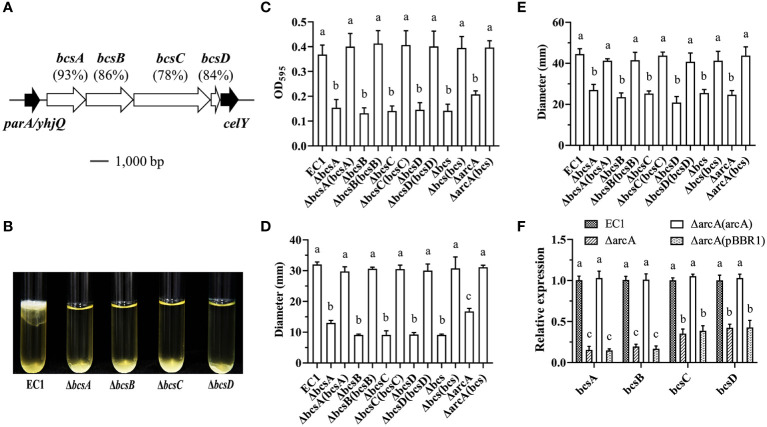
ArcA affects bacterial motility and biofilm formation by regulating the synthesis of celluloses. **(A)** Analysis of the homology and the locus of the cellulose encoding genes *bcsABCD* in *D*. *oryzae* EC1. **(B)** The qualitative analysis of biofilm formation in mutants of *bcsA*, *bcsB*, *bcsC* and *bcsD*. **(C)** Quantification of adherent biofilm biomass in wild-type strain EC1, ΔbcsA, ΔbcsB, ΔbcsC, ΔbcsD, Δbcs and ΔarcA(bcs) using crystal violet staining. **(D)** The swimming motility of wild-type strain EC1, ΔbcsA, ΔbcsB, ΔbcsC, ΔbcsD, Δbcs and ΔarcA(bcs). **(E)** The swarming motility of wild-type strain EC1, ΔbcsA, ΔbcsB, ΔbcsC, ΔbcsD, Δbcs and ΔarcA(bcs). **(F)** The gene expression of *bcsA*, *bcsB*, *bcsC* and *bcsD* in strains ΔarcA and ΔarcA(pBBR1). The experiments were repeated three times in triplicate and the error bars indicate standard deviation. The statistical analysis was performed on each group of data, and significant differences values (analysis of variance, *p* < 0.05) are indicated by different letters.

In *D. dadantii* 3937, the cellulose synthesis cluster *bcs* (containing *bcsA*, *bcsB*, *bcsC* and *bcsD*) plays a crucial role in biofilm formation (Prigent-Combaret et al., 2012). Bioinformatics analysis showed that in *D. oryzae* strain EC1, genes of *W909_RS19085*, *W909_RS19080*, *W909_RS19075* and *W909_RS19070* are homogenous to the *bcs* operon genes *bcsA*, *bcsB*, *bcsC* and *bcsD* in *D. dadantii* 3937, sharing 92.96%, 85.90%, 77.69% and 83.87% identities at amino acid level, respectively (**Figure 4A**). To determine the contribution of the *bcs* genes in biofilm formation, we deleted each gene and tested their non-adherent and attached biofilms. The result showed that all the *bcs* deletion mutants were reduced in biofilm formation at the air/liquid interface (**Figure 4B**) or substantially reduced in attachment to tuber walls (**Figure 4C**). Similarly, all mutants of *bcsA*, *bcsB*, *bcsC*, *bcsD* and deletion of the entire *bcs* operon significantly decreased swimming and swarming motility (**Figures 4D, E**). *In trans* expression of *bcs* genes in corresponding mutants restored the swimming and swarming motility to the wild-type level (**Figures 4D, E**).

To understand whether ArcBA regulates the expression of *bcsABCD*, their expression levels in wild-type strain EC1, ΔarcA, ΔarcA(pBBR1) and ΔarcA(arcA) were determined by RT-qPCR in LB medium at OD_600_ = 1.0. The results showed that the transcript level of *bcsA*, *bcsB*, *bcsC* and *bcsD* were decreased by 6.5-, 5.1-, 2.8- and 2.4-fold in ΔarcA, respectively (**Figure 4F**), and were decreased by 6.7-, 5.9-, 2.6- and 2.3-fold in ΔarcA(pBBR1), respectively (**Figure 4F**).

The above results imply that ArcBA might modulate biofilm formation and cell motility through its influence on the synthesis of cellulose. To test this deduction, we transferred the expression construct carrying the wild-type *bcs* gene operon consisting of *bcsA*, *bcsB*, *bcsC*, and *bcsD* to the ΔarcA mutant and conducted phenotype analysis. The results indicated that expression of the *bcs* operon in the mutant ΔarcA restored its biofilm biomass, swimming and swarming motility to the wild-type level (**Figures 4C–E**).

### Deletion of *arcB* and *arcA* decreased the virulence of *D. oryzae* EC1

**Figure 5:**
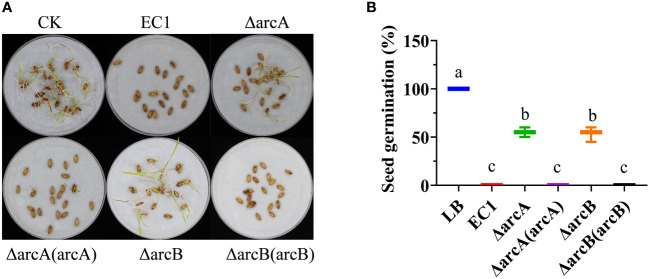
The *arcA* and *arcB* deletion mutants showed reduced pathogenicity and ability of infection on rice seeds. **(A)** Rice seed germination inoculated with strain EC1 and its derivatives. Twenty rice seeds were treated with EC1 and its derivatives, respectively, and the experiment was repeated twice. **(B)** Germination rates of rice seeds treated with strain EC1 and its derivatives. Statistical analysis was performed on each group of data and significantly different values (analysis of variance, *p* < 0.05) are indicated by different letters.

*D. oryzae* strain EC1 can inhibit rice seed germination, even at a low cell density (Hussain et al., 2008; Zhou et al., 2016). To determine the virulence of ΔarcA and ΔarcB, we treated rice seeds with 10^2^ bacterial cells. The rice seed germination rate was determined one week after incubation at 27°C. The results showed that about 60% of rice seeds treated with the mutants ΔarcA and ΔarcB were germinated (**Figures 5A, B**). In contrast, no germination was observed for the rice seeds challenged with wild type strain EC1 or the complemented strains ΔarcA(arcA) and ΔarcB(arcB), respectively (**Figures 5A, B**). Given that ArcBA could positively regulate the bacterial motility (**Figure 3**), we speculated that null mutation of this TCS might compromise the bacterial activity in invasion and systemic infection. To verify this possibility, the *gfp* gene was cloned under the control of the *lac* promoter in the vector pLAFR3, which was introduced into strain EC1 and mutant ΔarcA to generate the corresponding fluorescence tagged strains. The inoculated rice seeds were incubated at 28 °C for 24 h before removing the seed husk and observation of bacterial invasion under a fluorescence microscope. The results showed that deletion of *arcA* reduced the bacterial ability to invade and infect rice seeds (**Figure S5**).

### ArcA modulates the expression of the key virulence regulators Fis, SlyA and OhrR

**Figure 6:**
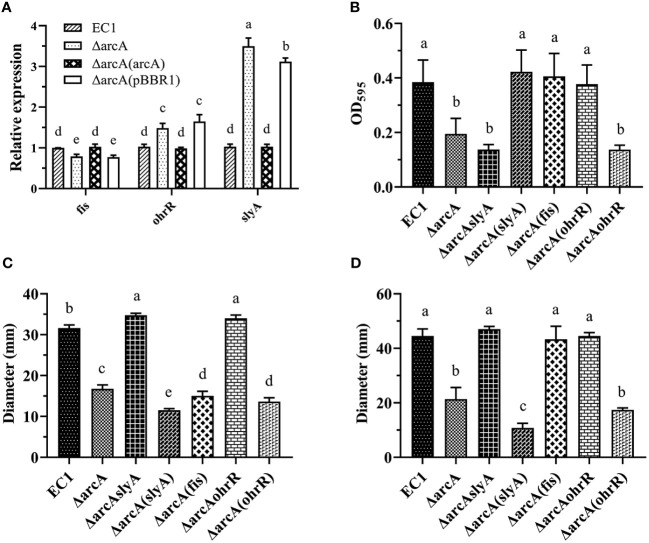
ArcA regulates the expression of *fis* positively, and *slyA* and *ohrR* negatively. **(A)** The expression of *fis*, *slyA*, and *ohrR* in the *arcA* deletion mutant. **(B)** The biofilm formation by wild-type strain EC1, ΔarcA, ΔarcAslyA, ΔarcA(slyA), ΔarcA(fis) and ΔarcA(ohrR). **(C)** Swimming motility of wild-type strain EC1, ΔarcA, ΔarcAslyA, ΔarcA(slyA), ΔarcA(fis) and ΔarcA(ohrR). **(D)** Swarming motility of wild-type strain EC1, ΔarcA, ΔarcAslyA, ΔarcA(slyA), ΔarcA(fis) and ΔarcA(ohrR). The experiments were repeated three times in triplicate. Statistical analysis was performed on each group of data and significantly different values (analysis of variance, *p* < 0.05) are indicated by different letters.

Our previous studies showed that several transcriptional regulators, i.e., Fis, SlyA and OhrR, play crucial roles in modulation of biofilm formation and bacterial motility in *D. oryzae* EC1 (Zhou et al., 2016; Lv et al., 2018; Lv et al., 2022). Interestingly, these regulators seem to affect the bacterial motility in opposite ways. We found that Fis could positively modulate the bacterial biofilm formation and cell motility (Lv et al., 2018), whereas SlyA and OhrR act by positively regulating biofilm formation but negatively regulating swimming and swarming motility (Zhou et al., 2016; Lv et al., 2022). To understand the relationships between ArcA and these regulators in the bacterial regulatory network, the expression levels of the genes *fis*, *slyA* and *ohrR* in the *arcA* mutant were determined by RT-qPCR. The results showed that the transcript level of *fis* was decreased by about 1.3-fold, whereas the expression level of *slyA* and *ohrR* were increased by about 3.3- and 1.5-fold, respectively, in the *arcA* mutant compared with the wild type EC1 (**Figure 6A**).

*In trans* expression of *fis*, *slyA* and *ohrR*, respectively, in the *arcA* mutant rescued the biofilm formation to the wild-type level (**Figure 6B**), agreeable with their positive regulatory roles in modulation of biofilm formation (Zhou et al., 2016; Lv et al., 2018; Lv et al., 2022) (**Figure 8**). *In trans* expression of the positive motility regulator gene *fis* in the *arcA* mutant rescued its bacterial swarming motility as expected, but somehow it failed to restore the swimming motility to the wild-type level (**Figures 6C, D**). Similarly, as expected, *in trans* expression of the negative motility regulatory genes *slyA* and *ohrR*, respectively, in the *arcA* mutant further dampened down the bacterial swimming and swarming motility compared with the *arcA* mutant.

Given that deletion of the motility positive regulator gene *arcA* led to increased expression of *slyA* and *ohrR* (**Figure 6A**), which play a negative role in modulation of the bacterial motility (Zhou et al., 2016; Lv et al., 2022) (**Figure 8**), we thought that increased expression of *slyA* and *ohrR* in the mutant ΔarcA might account for the decreased bacterial motility. To test this hypothesis, we generated the double deletion mutants ΔarcAslyA and ΔarcAohrR, respectively, and examined their phenotype changes. The results showed that deletion of either *slyA* or *ohrR* in the mutant ΔarcA could recover swimming and swarming motility to the wild-type level (**Figures 6C, D**).

**Figure 7:**
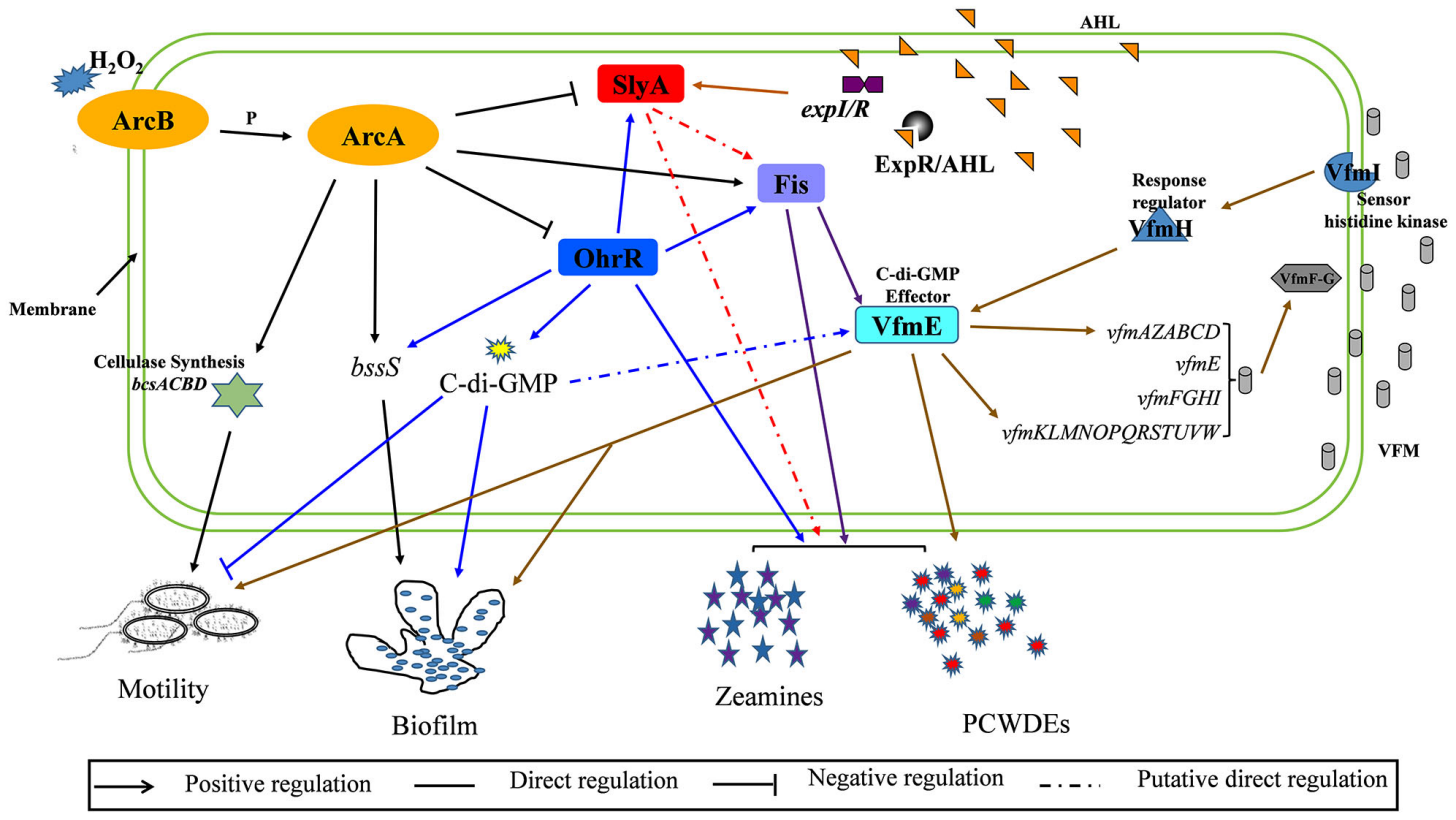
A schematic map of ArcBA modulating downstream genes in *Dickeya oryzae* EC1. ArcA modulates bacterial motility and biofilm formation by directly binding to the promoter region of *bcsA*. ArcA also directly and positively regulates the transcription of *fis*, and negatively regulates the transcription of *slyA* and *ohrR*.

In view of previous findings that deletion of the MarR family transcriptional regulators SlyA and OhrR significantly decreased biofilm formation and enhanced cell motility, deletion of transcriptional regulator Fis markedly reduced biofilm formation and cell motility, and OhrR modulated the transcription of SlyA and Fis through binding to the promoters of *slyA* and *fis* in *D. oryzae* (Zhou et al., 2016; Lv et al., 2018; Lv et al., 2022). This study results demonstrated that ArcBA modulates cell motility and biofilm formation through positive regulation on the synthesis of cellulose and the expression of *fis*, and negative regulation on the expression of *slyA* and *ohrR* (**Figure 7**), as well, the production of zeamines and PCWDEs was modulated by a variety of complex regulatory pathway constituted by Fis, SlyA and OhrR (**Figure 7**).

### ArcA directly interacts with the promoters of a range of virulence genes

**Figure 8:**
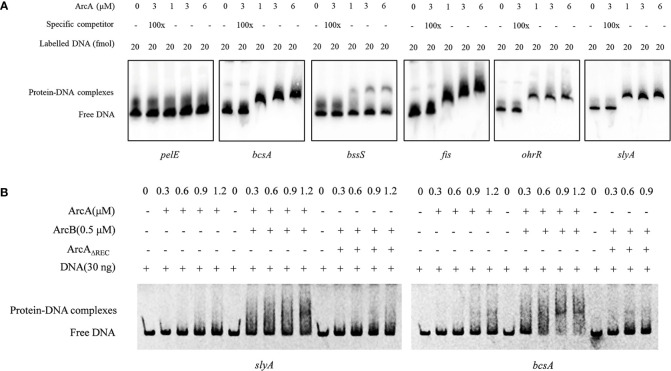
Interaction of ArcA with target genes. **(A)** Labeled DNA sequences of 20 fmol corresponding promoters of *pelE*, *bcsA*, *zmsA*, *bssS*, *fis*, *slyA* and *ohrR* were incubated with 1 μM, 3 μM, and 6 μM ArcA protein, respectively. About 100-fold higher amount of unlabeled corresponding promoter DNAs were used as specific competitors. The positions of protein-DNA complexes and free DNA probes were shown. **(B)** Binding ability of ArcA with target probes is enhanced by addition of ArcB and reduced after deletion of the REC domain. The experiment was performed at least twice with similar results.

To further understand the regulatory mechanisms of ArcA, we carried out electrophoretic mobility shift assay (EMSA) using purified ArcA protein and the DNA fragments corresponding to the promoter regions of putative target genes of ArcA. The results showed that ArcA could interact with the promoters of *bcsA*, *bssS*, *fis*, *slyA* and *ohrR*, indicating that ArcA could directly modulate the transcription of these genes, and thus regulate the biofilm formation and swimming and swarming motility (**Figure 8A**). Consistent with the finding that deletion of *arcA* did not affect the expression of PCWDEs (**Figure S3**), ArcA could not bind to the promoter of *pelE* which encodes a pectate lyase (**Figure 8A**).

Previous studies on sensor kinases, including ArcB, showed that removal of the transmembrane segments does not affect the processes of autophosphorylation and the subsequent transphosphorylation of the cognate regulator proteins (Pena-Sandoval and Georgellis, 2010). To determine whether ArcB can affect the activity of ArcA through the processes of autophosphorylation, we examined the binding ability of ArcA to the promoter sequences of target genes under conditions after addition of ArcB. The results showed that addition of ArcB enhanced the affinity of ArcA to the promoters of *bcsA* and *slyA* (**Figure 8B**), indicating the interaction between ArcB and ArcA. Furthermore, deletion of the REC domain in the ArcA protein reduced the binding ability to the promoters of target genes (**Figure 8B**), demonstrating the importance of phosphorylation of ArcA in gene regulation.

## Discussion

It is well known that motility and biofilm are important virulence determinants and survival strategies to cope with harsh environmental conditions and infect hosts for pathogenic bacteria (Mah, 2012; Nakane, 2015; Olsen, 2015; Del Pozo, 2018; Nakamura, 2019). In this study, we identified a TCS of ArcBA that exhibits a conservative profile at the amino acid level with the homolog in *E. coli* (**Figure S1**), and systematically elucidated the roles of ArcBA in regulation of biofilm formation, cell motility, oxidative stress resistance, and the virulence in *D. oryzae* EC1.

Previous studies have unveiled that the Arc (aerobic respiratory control) TCS is a key regulatory system facilitating facultative anaerobic bacteria to sense and respond to various respiratory growth conditions and regulate their gene expression accordingly, such as *Salmonella enterica* serovar *Typhimurium*, *E. coli*, *Haemophilus parasuis* serova 13 clincal strain EP3, and *S. enteritidis* (Gao et al., 2007; Calderon et al., 2011; Cameron et al., 2013; Jiang et al., 2015; Ding et al., 2016; Basan et al., 2017; Chen and Goulian, 2018; Fernandez et al., 2018; Pardo-Este et al., 2019), as well as regulating virulence factor production, controlling metabolism, chemotaxis, motility and pathogenicity of avian pathogenic *E. coli* (Jiang et al., 2015), virulence of *H. parasuis* serovar 13 clinical strain EP3 (Ding et al., 2016), and swarming motility in *Serratia marcescens* FS14 (Zhang et al., 2018). Similarly, in this study, we demonstrated that ArcBA regulates the virulence traits, bacterial swimming and swarming motility, and gene expression of flagellar in *D. oryzae* (**Figures 3A–C**). Furthermore, deletion of *arcA* and *arcB* markedly decreased biofilm formation (**Figure 1**), resistance to hydrogen peroxide (**Figure 2**), and survival at the stationary phase (**Figure S2**). It is possible that these phenotypic traits play an important role in colonization and survival in host plants when hydrogen peroxide bursts during infection by pathogenic bacteria.

Cellulose is a key component of plant cell walls and the most abundant biopolymer on earth. Most celluloses are produced by plant cellulose synthase complexes, which originated from bacteria (Nobles et al., 2001; Nobles and Brown, 2004) and have been found in a variety of bacteria (Ross et al., 1991; Jahn et al., 2011). Cellulose and its derivatives have been characterized as important extracellular matrix components of biofilms and play a key role in the regulation of virulence in important pathogenic bacteria (Spiers et al., 2003; Romling et al., 2013). The *bcsABCD* operon has been demonstrated to be involved in cellulose biosynthesis, export and packaging of dextran molecules (Saxena et al., 1990; Wong et al., 1990; Omadjela et al., 2013). Previous studies have revealed that cellulose synthase BcsA inhibits the ability of *S. typhimurium* to bind to and invade the bacterium *Acanthamoeba castellanii* (Gill et al., 2018). Pathogenic bacteria, such as *E. coli* and *Salmonella* produce extracellular celluloses, which have been proven to be involved in biofilm formation and host colonization. The genes *bcsA* and *bcsB* of *Cronobacter* species are necessary for the production of celluloses and are involved in the formation of biofilms and cell aggregations (Hu et al., 2015). In *D. dadantii*, the production of celluloses is necessary for the formation of pellicle-biofilms and resistance to chlorine treatment. The expression of the *bcs* operon is regulated by the growth stage, stimulated in the biofilms, and inhibited by a global regulator Fis directly through interacting with the *bcs* promoter (Jahn et al., 2011; Prigent-Combaret et al., 2012). Similarly, *bssS* regulates the biofilm through regulating quorum sensing signal section in *E. coli* (Domka et al., 2006). In *D. oryzae*, *bssS* encodes a biofilm formation regulatory protein that plays an important role in modulation of biofilm formation (Lv et al., 2022). In this study, the biofilm formation of the deletion mutants ΔbcsA, ΔbcsB, ΔbcsC, ΔbcsD and Δbcs were abolished completely (**Figure 5B**). The abilities of motility and biofilm formation were restored by transformation with plasmids carrying the wild-type *bcs* genes into the mutant of ΔarcA (**Figures 4C–E**). The RT-qPCR and EMSA analyses strongly suggest that ArcBA influences the biofilm formation and cell motility mainly through direct interaction with the promoters of the *bcs* cellulose synthesis genes and *bssS* (**Figures 4F**, **8** and **S7**). However, it is worth further study on the regulatory mechanism of *bssS*, as well as its relationship with the *bcs* cellulose synthesis gene cluster in *D. oryzae*.

Similar to ArcA, Fis, SlyA and OhrR are three global regulators modulating biofilm formation and cell motility in *D. oryzae* (Zhou et al., 2016; Lv et al., 2018; Lv et al., 2022). In this study, we demonstrated that ArcA modulates biofilm formation and cell motility by direct interaction with Fis, SlyA and OhrR, in which, positive action on Fis and negative action on SlyA and OhrR (**Figure 7**). Alternatively, ArcA also binds to the promoter of *bcs* operon and *bssS* to directly affect biofilm formation and motility (**Figure 7**). Our previous study revealed the similar patterns of OhrR and SlyA in regulating biofilm formation and motility, and the direct binding of OhrR to *slyA* promoter (Lv et al., 2022), implicating that the pathway of ArcBA(-OhrR)-SlyA regulating on biofilm formation and motility. Furthermore, a recent study identified VfmE as the c-di-GMP effector (Banerjee et al., 2022), and our previous study showed the direct interaction between OhrR and Fis, and between Fis and VfmE (Lv et al., 2018; Lv et al., 2022), thus, another pathway regulating biofilm formation and motility is the ArcBA(-OhrR)-Fis-VfmE (**Figure 7**). In addition, our results showed that the ArcBA TCS did not affect the production of PCWDEs and zeamines (**Figures S3** and **S4**), however, EMSA showed that ArcA could directly interact with the promoter of *zmsA* (**Figure S6**), which contributes to the synthesis of zeamines (Zhou et al., 2015). One of the reasons for this contradiction may be that the production of zeamines is controlled by a variety of complex regulatory pathways, such as ArcA, Fis, SlyA and OhrR (**Figure 7**), and the influence of ArcA on zeamine production could be recovered by that of SlyA and OhrR.

In addition, several other regulators have been demonstrated in other species of *Dickeya* to control the production of virulence factors, whereas, their roles have not been examined yet in *D. oryzae* EC1, for example, a newly identified MarR family transcriptional regulator MfbR activating genes encoding PCWDEs in *D. dadantii* 3937 (Reverchon et al., 2010) and regulators PecS, PecT, KdgR, and H-NS are known to be associated with the regulation of the production of PCWDEs (Yang et al., 2008; Reverchon et al., 2010; Morales et al., 2013). Furthermore, quorum sensing systems have also been demonstrated to contribute to cell motility, biofilm formation and virulence in *D. oryzae*. For instance, AHL regulates biofilm formation positively and cell motility negatively (Hussain et al., 2008), the VfmHI TCS as a receptor for Vfm quorum sensing signal positively regulates biofilm formation and cell motility (Lv et al., 2019), putrescine serves as an intraspecies and interkingdom cell-cell communication signal modulating biofilm formation and cell motility (Shi et al., 2019). Therefore, it is meaningful to investigate the association of ArcBA with these regulators, and further elaborate the virulence regulatory network in *D. oryzae* EC1.

In summary, the results of this study demonstrated that TCS ArcBA plays a crucial role in virulence traits in *D. oryzae* EC1, including cell motility, biofilm formation and infection on rice seeds. In particular, we showed that TCS ArcBA modulates the synthesis of celluloses to alter cell motility and biofilm formation. Meanwhile, we showed that ArcA positively regulates the global transcriptional regulator Fis and negatively regulates SlyA and OhrR global transcriptional regulators in *D. oryzae*. These finding would help us to better understand the complex regulatory mechanism that modulates the physiology and virulence of *D. oryzae*.

## Data availability statement

The raw data supporting the conclusions of this article will be made available by the authors, without undue reservation.

## Author contributions

ML and LZ conceived the study. JZ and XZ supervised the study. ML, SY, MH, YX, and ZL performed the experiments. SY, ML, and XZ analyzed the data. ML and SY drafted the manuscript. JZ and LZ revised the manuscript. All authors contributed to the revisions.

## Funding

This work was supported by the grants from the National Natural Science Foundation of China (31901843 and 31972230), the Key-Area Research and Development Program of Guangdong Province (2020B0202090001 and 2018B020205003) and the Natural Science Foundation of Guangdong Province, China (2020A1515011534).

## Conflict of interest

The authors declare that the research was conducted in the absence of any commercial or financial relationships that could be construed as a potential conflict of interest.

## Publisher’s note

All claims expressed in this article are solely those of the authors and do not necessarily represent those of their affiliated organizations, or those of the publisher, the editors and the reviewers. Any product that may be evaluated in this article, or claim that may be made by its manufacturer, is not guaranteed or endorsed by the publisher.

## Supplementary material

Supplementary Figure 1 The amino acid sequences of the two-component system proteins and physical map of ArcA and ArcB. **(A)** Sequences alignments of ArcA. **(B)** Sequences alignments of ArcB. **(C)** Domain organization of the ArcA and ArcB proteins was analyzed based on predictions of Protein families’ database of alignments (Pfam) and Simple Modular Architecture Research Tool (SMART). This two-component phosphotransfer system consists of a dimeric transmembrane sensor hisditine kinase (HK) and a cytoplasmic response regulator (RR). TM1 and TM2 indicate the location of the transmembrane segments and ArcB (HK) is composed of PAS domain, PAC domain, HisKA domain, HATPase_c domain, REC domain and HTP domain. The ArcA (RR) contains two function domains of REC and Trans_reg_c.

Supplementary Figure 2 A growth kinetic analysis of strain EC1 and its derivatives. **(A)** A growth kinetics determination of strain EC1 and its derivatives in LB medium. **(B)** A growth kinetics determination of strain EC1 and its derivatives in SOBG medium. The wild-type strain EC1 and its derivative strains showed similar growth curves at exponential growth phase, both ΔarcB and ΔarcA significantly reduced the ability to survival than EC1 and their complementary strains at stationary phase and decline phases in LB and SOBG medium. Experiments were performed three times and in triplicates.

Supplementary Figure 3 Effect of *arcA* and *arcB* deletion on cellulase, protease, pectase and polygalacturonase production in *Dickeya oryzae* EC1. Enzymatic activities were measured for cellulases **(A)**, proteases **(B)**, pectases **(C)** and polygalacturonases **(D)** using the assay plates with corresponding substrates. The experiment was repeated three times in triplicate, and error bars represent standard deviations. Statistical analysis was performed on each group of data and significantly different values (analysis of variance, *p* < 0.05) are indicated by different letters.

Supplementary Figure 4 Effect of *arcA* and *arcB* deletion on zeamine production in *Dickeya oryzae* EC1. **(A)** quantitative determination of zeamine production by strain EC1 and its derivatives. The concentration of zeamines was determined by this formula: zeamines (unit) = 0.5484e^0.886^ *^x^* (*R* ^2^ = 0.9957), *x* is the radius in mm of the inhibition zone surrounding the well. For comparison, the data of *arcA* and *arcB* mutants and their complemented strains were normalized to that of the wild-type EC1, which was set to a value of 100%. The experiments were repeated three times in triplicates. Statistical analysis was performed on each group of data and significantly different values (analysis of variance, *p* < 0.05) are marked with different letters. **(B)** Qualitative detection of zeamine production by strain EC1 and its derivative strains. The antimicrobial activity bioassay plates were prepared by pouring 20 ml of 1% agarose containing 10^8^ cells of *E. coli* DH5α on 15 ml solid LB agar.

Supplementary Figure 5 ArcA directly interacts with the promoter region of *zmsA*. Labeled DNA sequence of 20 fmol promoter of *zmsA* was incubated with 1 μM, 3 μM and 6 μM ArcA, respectively. About 100-fold higher amount of unlabeled *zmsA* promoter DNA was used as the specific competitor. The positions of protein-DNA complexes and free DNA probe were shown. The experiment was performed twice with similar results.

Supplementary Figure 6 The *arcA* deletion mutant showed reduced ability of infection on rice seeds. Twenty rice seeds were treated with the 10^2^ cfu *gfp*-labeled strains of EC1 and ΔarcA at room temperature for 5 h, respectively, and then transferred onto moistened filter papers in plates. The husks of rice seeds were moved after the rice seeds incubation at 28 °C for 16-light and 8-h dark condition and examined under a fluorescence microscope. Rice seeds treated with sterilized water were set as a blank control.

Supplementary Figure 7 The gene expression of *bssS* detection. The gene expression of *bssS* in *arcA* deletion mutant and wild-type strain EC1. The experiment was repeated three times in triplicate. *****p* < 0.0001, Student’s *t* test.
