# Supplementary figures and images for: Two-component system ArcBA modulates cell motility and biofilm formation in Dickeya oryzae
Source: Front Plant Sci. 2022 Oct 21;13:1033192. doi: 10.3389/fpls.2022.1033192 (PMC9634086; doi:10.3389/fpls.2022.1033192)

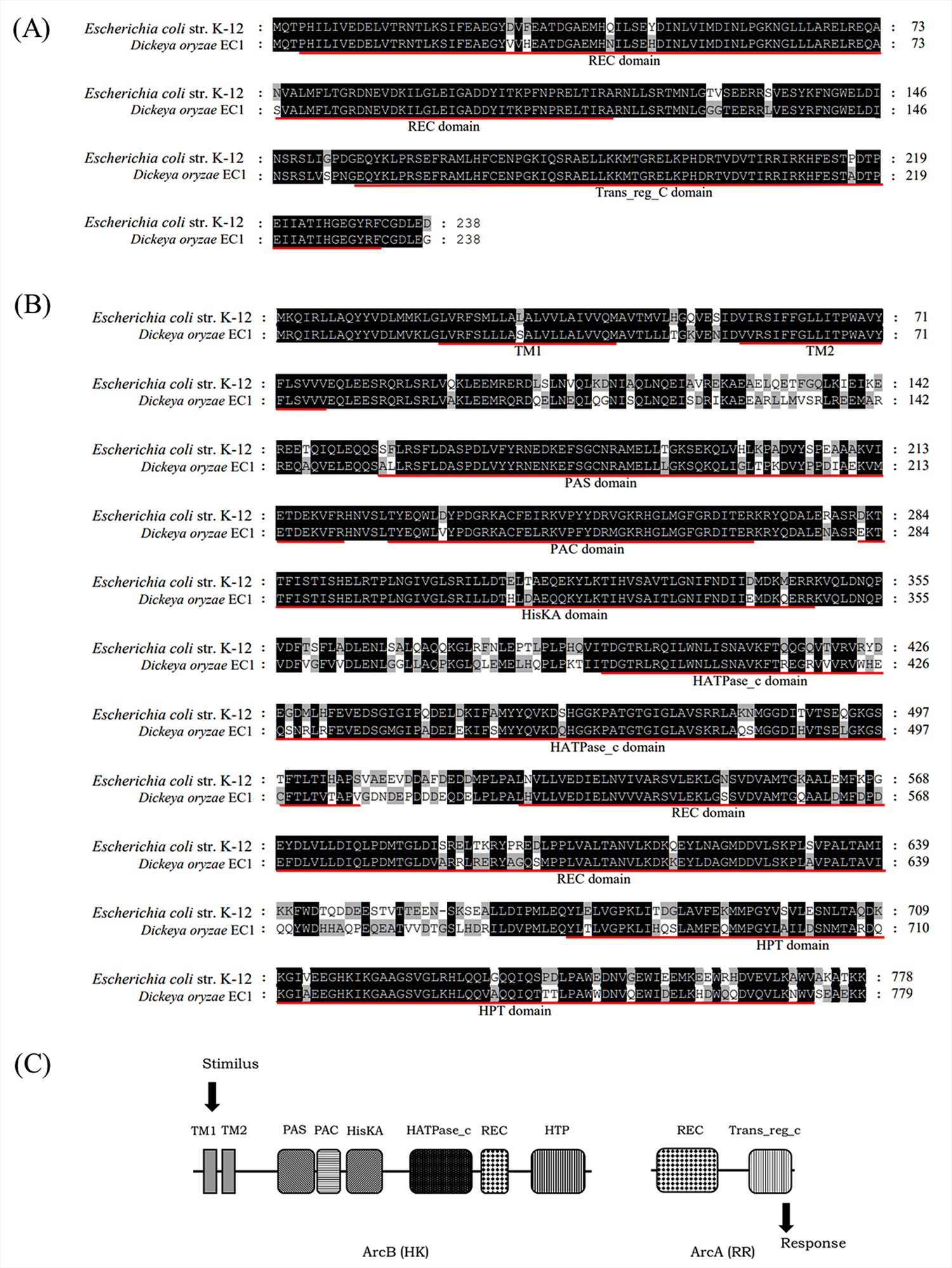

Supplement: Supplementary Figure 1 — The amino acid sequences of the two-component system proteins and physical map of ArcA and ArcB. (A) Sequences alignments of ArcA. (B) Sequences alignments of ArcB. (C) Domain organization of the ArcA and ArcB proteins was analyzed based on predictions of Protein families’ database of alignments (Pfam) and Simple Modular Architecture Research Tool (SMART). This two-component phosphotransfer system consists of a dimeric transmembrane sensor hisditine kinase (HK) and a cytoplasmic response regulator (RR). TM1 and TM2 indicate the location of the transmembrane segments and ArcB (HK) is composed of PAS domain, PAC domain, HisKA domain, HATPase_c domain, REC domain and HTP domain. The ArcA (RR) contains two function domains of REC and Trans_reg_c. [file Image_1.tif]

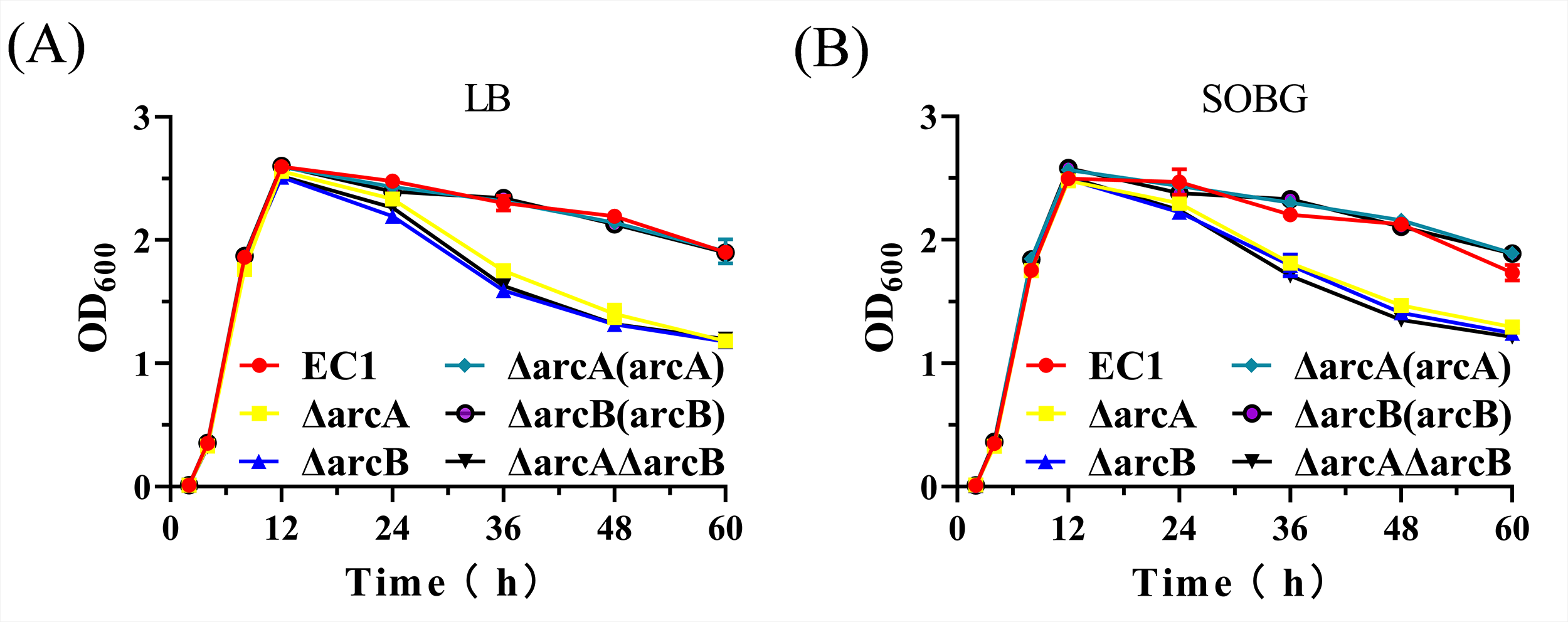

Supplement: Supplementary Figure 2 — A growth kinetic analysis of strain EC1 and its derivatives. (A) A growth kinetics determination of strain EC1 and its derivatives in LB medium. (B) A growth kinetics determination of strain EC1 and its derivatives in SOBG medium. The wild-type strain EC1 and its derivative strains showed similar growth curves at exponential growth phase, both ΔarcB and ΔarcA significantly reduced the ability to survival than EC1 and their complementary strains at stationary phase and decline phases in LB and SOBG medium. Experiments were performed three times and in triplicates. [file Image_2.tif]

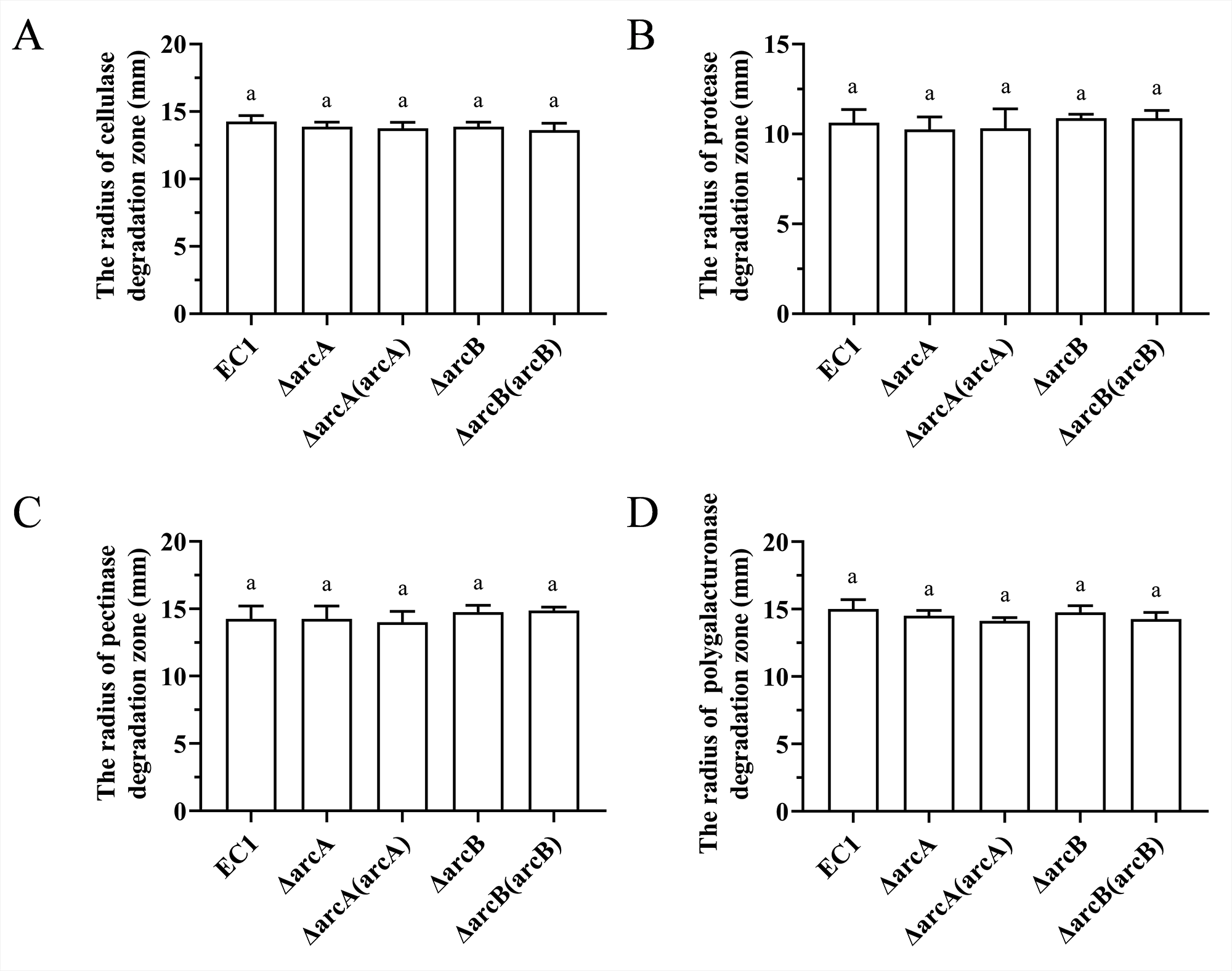

Supplement: Supplementary Figure 3 — Effect of arcA and arcB deletion on cellulase, protease, pectase and polygalacturonase production in Dickeya oryzae EC1. Enzymatic activities were measured for cellulases (A), proteases (B), pectases (C) and polygalacturonases (D) using the assay plates with corresponding substrates. The experiment was repeated three times in triplicate, and error bars represent standard deviations. Statistical analysis was performed on each group of data and significantly different values (analysis of variance, p < 0.05) are indicated by different letters. [file Image_3.tif]

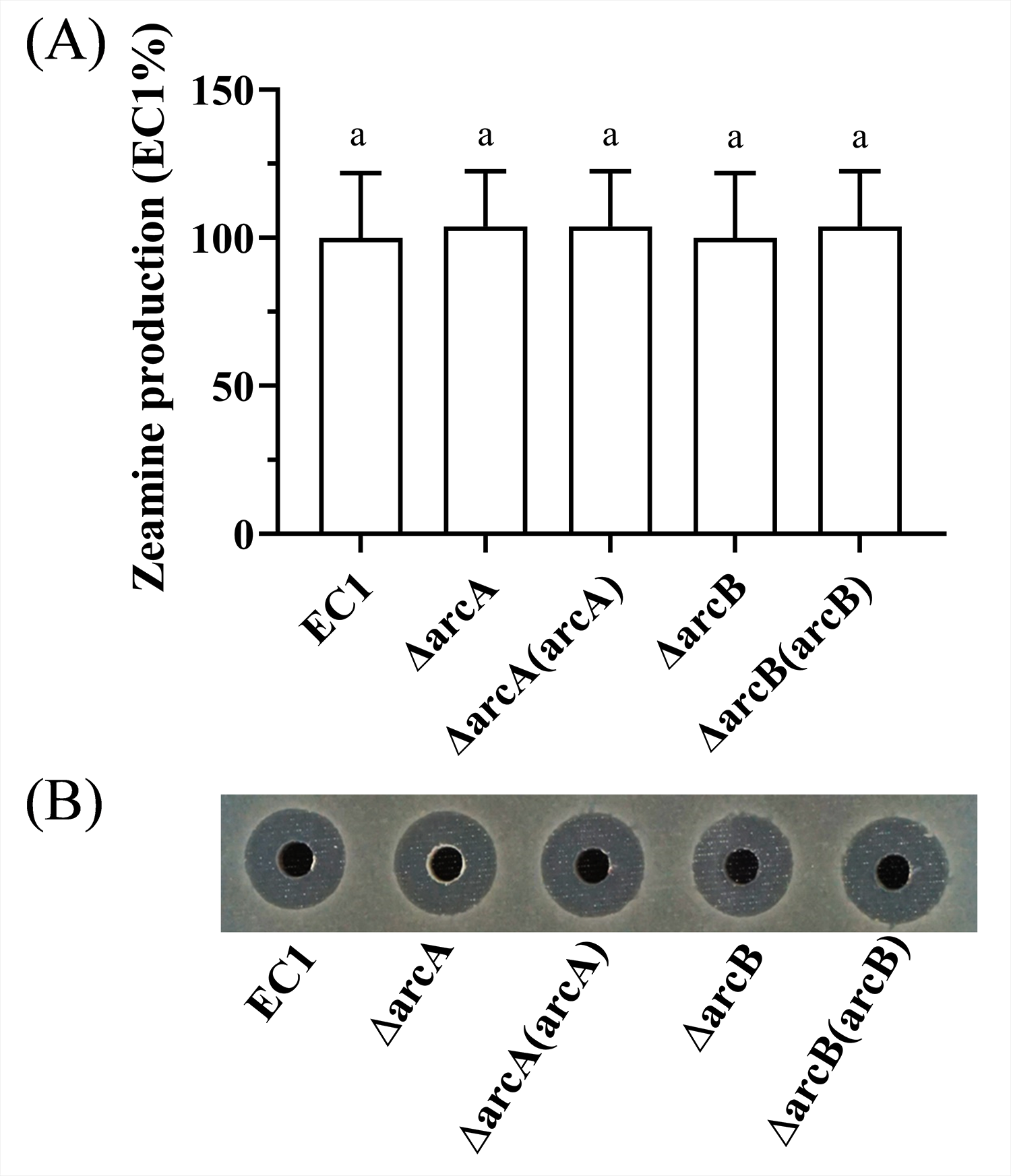

Supplement: Supplementary Figure 4 — Effect of arcA and arcB deletion on zeamine production in Dickeya oryzae EC1. (A) quantitative determination of zeamine production by strain EC1 and its derivatives. The concentration of zeamines was determined by this formula: zeamines (unit) = 0.5484e0.886 x (R 2 = 0.9957), x is the radius in mm of the inhibition zone surrounding the well. For comparison, the data of arcA and arcB mutants and their complemented strains were normalized to that of the wild-type EC1, which was set to a value of 100%. The experiments were repeated three times in triplicates. Statistical analysis was performed on each group of data and significantly different values (analysis of variance, p < 0.05) are marked with different letters. (B) Qualitative detection of zeamine production by strain EC1 and its derivative strains. The antimicrobial activity bioassay plates were prepared by pouring 20 ml of 1% agarose containing 108 cells of E. coli DH5α on 15 ml solid LB agar. [file Image_4.tif]

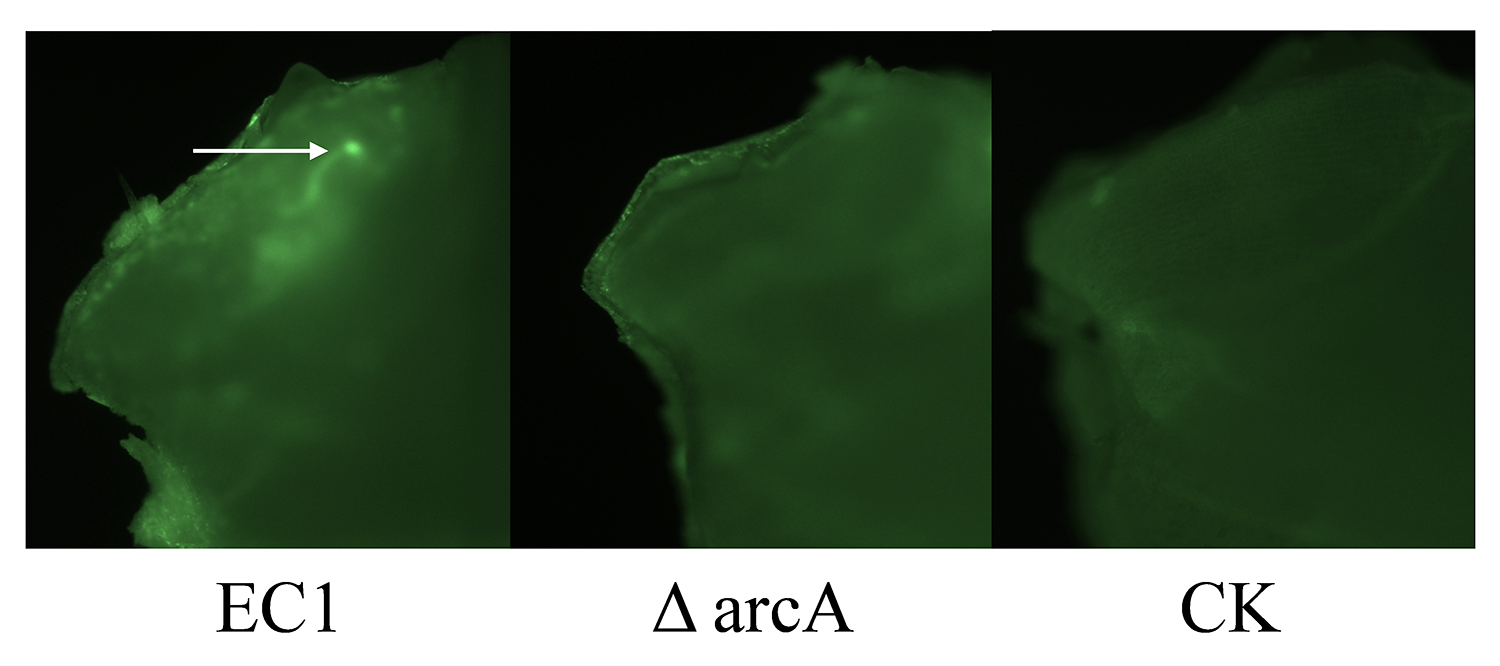

Supplement: Supplementary Figure 5 — ArcA directly interacts with the promoter region of zmsA. Labeled DNA sequence of 20 fmol promoter of zmsA was incubated with 1 μM, 3 μM and 6 μM ArcA, respectively. About 100-fold higher amount of unlabeled zmsA promoter DNA was used as the specific competitor. The positions of protein-DNA complexes and free DNA probe were shown. The experiment was performed twice with similar results. [file Image_5.tif]

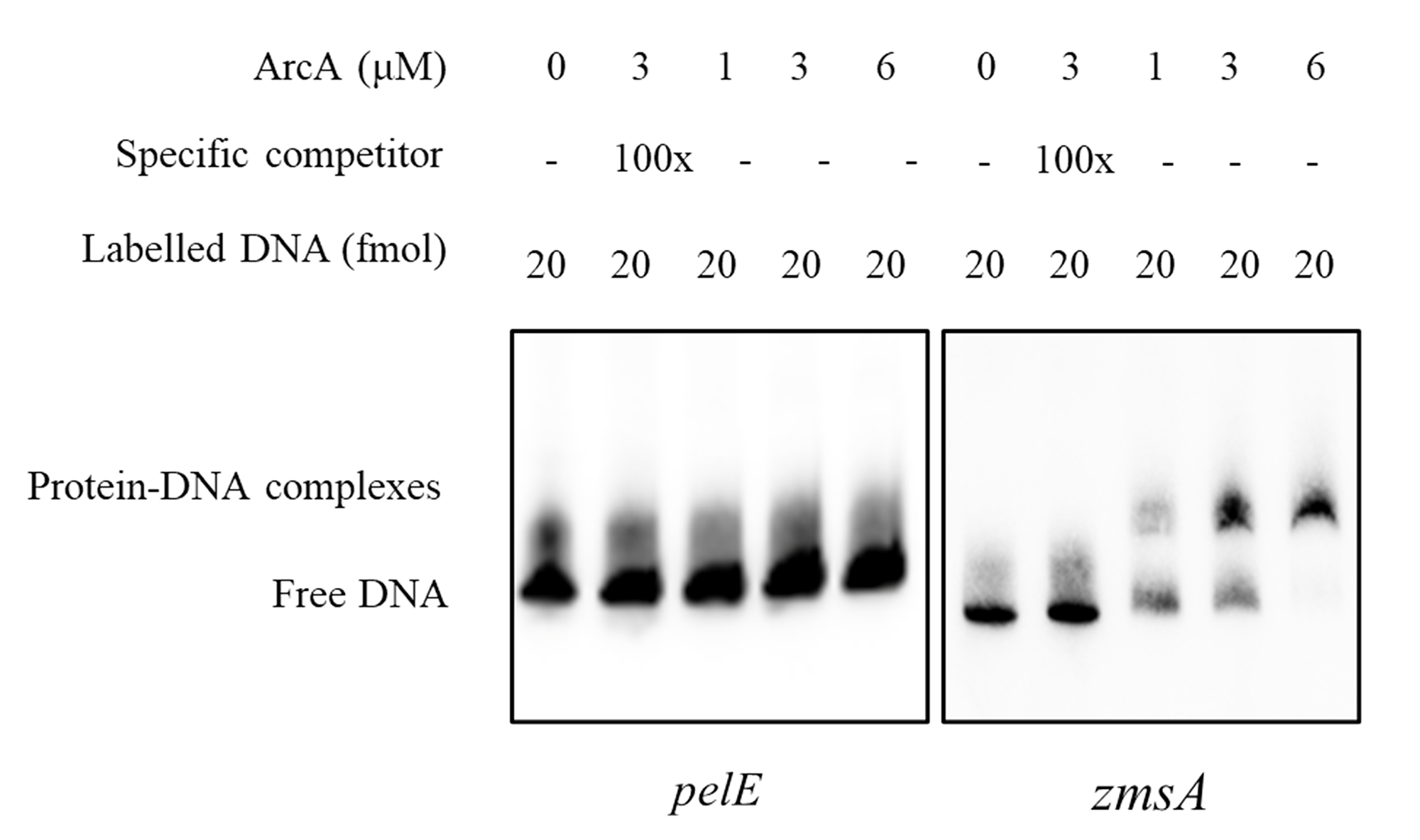

Supplement: Supplementary Figure 6 — The arcA deletion mutant showed reduced ability of infection on rice seeds. Twenty rice seeds were treated with the 102 cfu gfp-labeled strains of EC1 and ΔarcA at room temperature for 5 h, respectively, and then transferred onto moistened filter papers in plates. The husks of rice seeds were moved after the rice seeds incubation at 28 °C for 16-light and 8-h dark condition and examined under a fluorescence microscope. Rice seeds treated with sterilized water were set as a blank control. [file Image_6.tif]

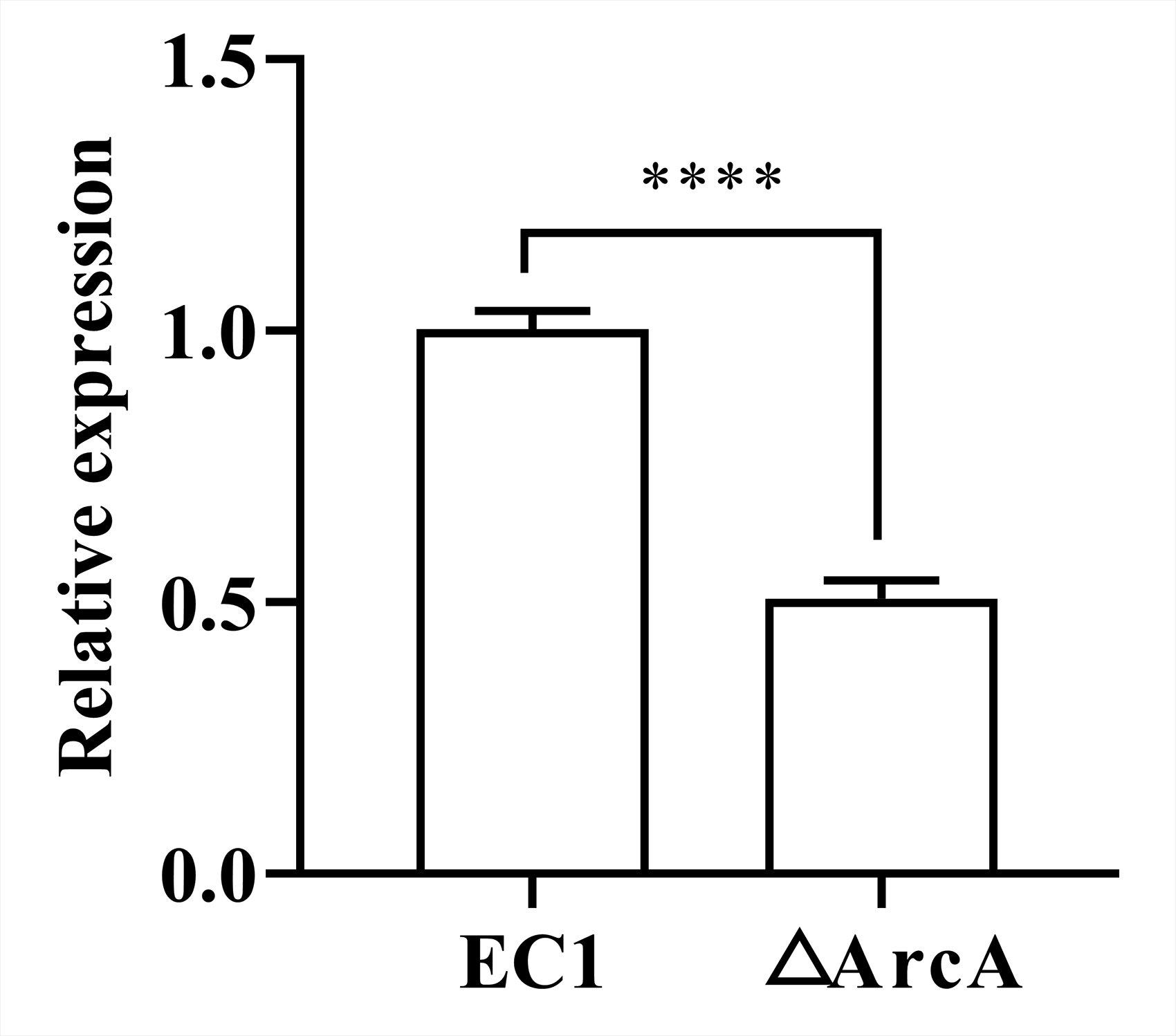

Supplement: Supplementary Figure 7 — The gene expression of bssS detection. The gene expression of bssS in arcA deletion mutant and wild-type strain EC1. The experiment was repeated three times in triplicate. ****p < 0.0001, Student’s t test. [file Image_7.tif]
