# Supplementary material for: Two-component system ArcBA modulates cell motility and biofilm formation in Dickeya oryzae
Source: Front Plant Sci. 2022 Oct 21;13:1033192. doi: 10.3389/fpls.2022.1033192 (PMC9634086; doi:10.3389/fpls.2022.1033192)
Supplement: Supplementary file 8 [file Table_1.docx]

**Table S1.** Primers used in this study

| Primers | Sequence (5'-3') | Purpose |
| --- | --- | --- |
| Mutant construction primers | | |
| arcA-F | CAATCCGCAAACGACCAAACCC | Identification of *arcA* mutant |
| arcA -R | GGTAAACGACGGGCTCAGATAG |  |
| arcA -1 | cg**ggatcc**CTGGCAGGTGCGCTGATAATTC | Amplification of *arcA* upstream sequence |
| arcA -2 | TCAACAACTCGGCACGTGACTGTATGTTATGCATCTCGGCGCCG |  |
| arcA -3 | CGGCGCCGAGATGCATAACATACAGTCACGTGCCGAGTTGTTGA | Amplification of *arcA* downstream sequence |
| arcA -4 | gg**actagt**GTTACCGCTTAATGGTCTTCGG |  |
| arcB -F | TAATGGAATGAACCTCGCGGA | Identification of *arcB* mutant |
| arcB -R | CGCGATGCAAATGAAGCCAAT |  |
| arcB -1 | cccctgcaggtcgacggatccCAAAAATTAATCAATATGTTTGGTGGG | Amplification of *arcB* upstream sequence |
| arcB -2 | tctgttgGTAATACTGCGCCAACAGCCTG |  |
| arcB -3 | ggcgcagtattacCAACAGATTCAGACCACGACATTAC | Amplification of *arcB* downstream sequence |
| arcB -4 | cggactatagactatactagtTGGATAGTGGATGGATTTTTCCG |  |
| bcsA-F | TTAAACGTGGCCTCAGTTCG | Identification of *bcsA* mutant |
| bcsA-R | CCTCCGGCAACGTCAAC |  |
| bcsA-1 | cccctgcaggtcgacggatccGGCCATGACGGAACTGGC | Amplification of *bcsA* upstream sequence |
| bcsA-2 | ggcgttatcatgcCATCGATTATCCTGATATTTAATTGATG |  |
| bcsA-3 | atcgatgGCATGATAACGCCACGTTTTG | Amplification of *bcsA* downstream sequence |
| bcsA-4 | cggactatagactatactagtGCCGTAGCCGTCCATCCG |  |
| bcsB-F | GCATTTCCAGTTTGGTTTGCC | Identification of *bcsB* mutant |
| bcsB-R | CGGACTATTGCCACCGGG |  |
| bcsB-1 | cccctgcaggtcgacggatccCTCAATATTATTCATGCCTCAGCG | Amplification of *bcsB* upstream sequence |
| bcsB-2 | tatccgttcccgcGACAAAACGTGGCGTTATCATG |  |
| bcsB-3 | ttttgtcGCGGGAACGGATAAAAAATAAG | Amplification of *bcsB* downstream sequence |
| bcsB-4 | cggactatagactatactagtCAATATTTGCAACCCTTCACGC |  |
| bcsC-F | TGACCGTGACCACATTGC | Identification of *bcsC* mutant |
| bcsC-R | CTGACCGAAGCGCATTT |  |
| bcsC-1 | cccctgcaggtcgacggatccTAAGACTCCGAGCATGTGGGC | Amplification of *bcsC* upstream sequence |
| bcsC-2 | tttaccCATACGTAGCTCGCTTATTTTTTATCC |  |
| bcsC-3 | gcgagctacgtatgGGTAAATAAGCATGAGTGACCTGC | Amplification of *bcsC* downstream sequence |
| bcsC-4 | cggactatagactatactagtGAGGGATTCAGGACCACATAACTG |  |
| bcsD-F | AGCCTGCTTTCTTATGTTGG | Identification of *bcsD* mutant |
| bcsD-R | GAGTAGTAATCTTCCGCAGC |  |
| bcsD-1 | cccctgcaggtcgacggatccAACGTACAACTGAGCTACGATAACG | Amplification of *bcsD* upstream sequence |
| bcsD-2 | ccggctcaatgCATGCTTATTTACCTCCGAGCC |  |
| bcsS-3 | ataagcatgCATTGAGCCGGGGGATAAC | Amplification of *bcsD* downstream sequence |
| bcsD-4 | cggactatagactatactagtCCGCCAGTAAGCCGCCCT |  |
| CarcA-HF | gtcgacggtatcgataagcttCATAACCTCCCAAACGACACCT | Amplification of *arcA* complementary sequence |
| CarcA-BR | cgctctagaactagtggatccGAATCAGCCTTCCAGATCACCG |  |
| Cfis-HF | CCCaagctt ATCCAGCGTTGGTCA | Amplification of *fis* complementary sequence |
| Cfis-BR | CGggatccAGGAGGCTCGTGTCTGT |  |
| CohrR-HF | CCCaagcttCCCTGATTGACGAGTCG | Amplification of *ohrR* complementary sequence |
| CohrR-BR | CGggatccCATGTGCAACGTAGAGAAC |  |
| MCS-F | GGCTCGTATGTTGTGTGG | Sequencing primers of pBBRI-MCS4 |
| MCS-R | AGCTGGCGTAATAGCGAAGA |  |
| pKNG-F | GACACTGAATACGGGGCAAC | Sequencing primers of pKNG 101 |
| pKNG-R | CCCCTGGATTTCACTGATGA |  |
| ArcA protein expression primers | | |
| pET32a-arcA-BamHI-F | gccatggctgatatcggatccATGCAGACCCCCCACATTCT | Prokaryotic expression of ArcA |
| pET32a-arcA-HindIII-R | ctcgagtgcggccgcaagcttTCAGCCTTCCAGATCACCGC |  |
| pET32a-arcB-BamHI-F | gccatggctgatatcggatccGAAGAGTCTCGCCAGCGCC | Prokaryotic expression of ArcB |
| pET32a-arcB-HindIII-R | ctcgagtgcggccgcaagcttTCATTTTTTTTCAGCCTCCGA |  |
| pET32a-arcA_∆REC_-F | gccatggctgatatcggatccCTGCTGTCCCGCACCATG | Prokaryotic expression of ArcA_∆REC_ |
| pET32a-arcA_∆REC_-R | ctcgagtgcggccgcaagcttTCAGCCTTCCAGATCACCGC |  |
| pET32a -F | TAATACGACTCACTATAGGG | Sequencing primers of pET32a |
| pET32a -R | GCTAGTTATTGCTCAGCGG |  |
| RT-qPCR primers | | |
| 16s1369F | CGGTGAATACGTTCYCGG | Reference gene of qRT-PCR |
| 16s1541R | AAGGAGGTGATCCRGCCGCA |  |
| ohrR-F | TGGCGATGAACAAGTTATATC | qRT-PCR of *ohrR* |
| ohrR -R | ACCAGCATCACCAGATAC |  |
| fis-F | AACTCTCAGGCTCAGGTA | qRT-PCR of *fis* |
| fis-R | CTCATACAGGTCATTCACATC |  |
| slyA-F | CAAAGGGTTAATTACCCGACA | qRT-PCR of *slyA* |
| slyA-R | ATCTAATATTTCGCCGCGTG |  |
| bcsA-F | TGACGCTGATACTGATTA | qRT-PCR of *bcsA* |
| bcsA-R | ACATAGAACTCGGCAATA |  |
| bcsB-F | AATCTGACGGTGAATAAG | qRT-PCR of *bcsB* |
| bcsB-R | TGACTCTTGATATTGTTGT |  |
| bcsC-F | CAATGGGATGATGGATAG | qRT-PCR of *bcsD* |
| bcsC-R | GGACAATACCTGATTCAT |  |
| bcsD-F | AGAATGTGAAGAGCAATA | qRT-PCR of *bssS* |
| bcsD-R | TGACCGTAACTGTAATAG |  |
| zmsA-F | CAGGATTATCAGTCAGTAGA | qRT-PCR of *zmsA* |
| zmsA-R | GTGCTCATTGCTATTCAG |  |
| bssS-F | TACCGTTGACAGTTATGA | qRT-PCR of *bssS* |
| bssS-R | GCCTCAAGGATAGAGATAA |  |
| FhlC-F | GCGATAAGCCTTGATAAC | qRT-PCR of *fhlC* |
| FhlC-R | GAACATCCACTCTTCCAT |  |
| FhlD-F | GGATTCTTGTGTTAATAGC | qRT-PCR of *fhlD* |
| FhlD-F | GGGTATCAATGAGGAAAT |  |
| FliA-F | CGCCACTCATCGTAAGAA | qRT-PCR of *fliA* |
| FliA-R | ACATCTCGTTAGAGGAATATCG |  |
| FliG-F | AAACGAACAGAAAGCCATT | qRT-PCR of *fliG* |
| FliG-R | TCACCACCACCGATAATC |  |
| FliM-F | ATCAAGATTCAGCCGTAT | qRT-PCR of *fliM* |
| FliM-F | CTTCAGGTGTATCAGGTT |  |
| DNA sequence amplification of promoters | | |
| zmsA-F | ATTCAGTGCTGCTGTGGTTTC | Amplification of *zmsA* promoter region sequence |
| zmsA-R | TCCGACAATTGCAATGTCACTTG |  |
| OhrR-F | CTGAACTGGGTGAGCGTCTG | Amplification of *ohrR* promoter region sequence |
| OhrR-R | CATGTGACTCCTTTGAGTGGATTATATTC |  |
| slyA-F | AGCCGACCCTGTTTTAATCG | Amplification of *slyA* promoter region sequence |
| slyA-R | CAACGAAGGCTGTTCTATTCCAATC |  |
| fis-F | GCAGAGTGTTTCCATTCCCG | Amplification of *fis* promoter region sequence |
| fis-R | AGTTCTGTCAGCTCTTTTTCTGTTTAC |  |
| bssS-F | TTATGCCGCCTCGAATAACC | Amplification of *bssS* promoter region sequence |
| bssS -R | CACAAGCGGGTGCGTTTG |  |
| bcsA-F | ACCACCGCAAAGGTTCTTAC | Amplification of *bcsA* promoter region sequence |
| bcsA-R | CATCGATTATCCTGATATTTAATTGATG |  |
| PelE-F | TTTCTGGTGACGCTGATTGC | Amplification of *pelE* promoter region sequence |
| PelE-R | CATCCTTGCAGCCCCCATAC |  |
| * The underlines are the site of restriction endonuclease | |  |
