# Supplementary material for: Two-component system ArcBA modulates cell motility and biofilm formation in Dickeya oryzae
Source: Front Plant Sci. 2022 Oct 21;13:1033192. doi: 10.3389/fpls.2022.1033192 (PMC9634086; doi:10.3389/fpls.2022.1033192)
Supplement: Supplementary file 9 [file Table_2.docx]

**Table S2.** The information of genes used to construct mutants for biofilm formation test in this study.

| Gene name | Protein_id | Gene locus | Biofilm formation (EC1%) | Function predicted |
| --- | --- | --- | --- | --- |
| SlmA | WP_012882897.1 | W909_RS00680 | 72.5 | nucleoid occlusion factor SlmA |
| MfbR | WP_016943684.1 | W909_RS03565 | 86.3 | MarR family transcriptional regulator |
| AcrR | WP_016942057.1 | W909_RS05150 | 116.5 | DNA-binding transcriptional repressor AcrR |
|  | WP_016941958.1 | W909_RS05735 | 87.9 | TetR/AcrR family transcriptional regulator |
| DctR | WP_016943817.1 | W909_RS06645 | 93.2 | DNA-binding response regulator |
| MfaR | WP_016943805.1 | W909_RS06705 | 109.6 | MarR family transcriptional regulator |
| Fnr | WP_016941395.1 | W909_RS09600 | 108.3 | transcriptional regulator FNR |
| **ArcA** | **WP_016940664.1** | **W909_RS16795** | **53.8** | **two-component system response regulator ArcA** |
